# Marine bacteria *Alteromonas* spp. require UDP-glucose-4-epimerase for aggregation and production of sticky exopolymer

**DOI:** 10.1128/mbio.00038-24

**Published:** 2024-07-03

**Authors:** Jacob M. Robertson, Erin A. Garza, Astrid K. M. Stubbusch, Christopher L. Dupont, Terence Hwa, Noelle A. Held

**Affiliations:** 1 Division of Biological Sciences, UC San Diego, La Jolla, California, USA; 2 Microbial and Environmental Genomics, J Craig Venter Institute, La Jolla, California, USA; 3 Department of Environmental Systems Science, Institute of Biogeochemistry and Pollutant Dynamics, ETH Zürich, Zürich, Switzerland; 4 Department of Environmental Microbiology, Eawag: Swiss Federal Institute of Aquatic Science and Technology, Dübendorf, Switzerland; 5 Department of Earth Sciences, Geological Institute, ETH Zurich, Zurich, Switzerland; 6 Department of Physics, UC San Diego, La Jolla, California, USA; 7 Department of Biological Sciences, Marine and Environmental Biology Section, University of Southern California, Los Angeles, California, USA; The University of Tennessee Knoxville, Knoxville, Tennessee, USA

**Keywords:** heterotrophic marine bacteria, aggregation, * galE*, marine snow, TEP

## Abstract

The physiology and ecology of particle-associated marine bacteria are of growing interest, but our knowledge of their aggregation behavior and mechanisms controlling their association with particles remains limited. We have found that a particle-associated isolate, *Alteromonas* sp. ALT199 strain 4B03, and the related type-strain *A. macleodii* 27126 both form large (>500 μm) aggregates while growing in rich medium. A non-clumping variant (NCV) of 4B03 spontaneously arose in the lab, and whole-genome sequencing revealed a partial deletion in the gene encoding UDP-glucose-4-epimerase (*galEΔ*308–324). In 27126, a knock-out of *galE* (*ΔgalE*::km^r^) resulted in a loss of aggregation, mimicking the NCV. Microscopic analysis shows that both 4B03 and 27126 rapidly form large aggregates, whereas their respective *galE* mutants remain primarily as single planktonic cells or clusters of a few cells. Strains 4B03 and 27126 also form aggregates with chitin particles, but their *galE* mutants do not. Alcian Blue staining shows that 4B03 and 27126 produce large transparent exopolymer particles (TEP), but their *galE* mutants are deficient in this regard. This study demonstrates the capabilities of cell-cell aggregation, aggregation of chitin particles, and production of TEP in strains of *Alteromonas*, a widespread particle-associated genus of heterotrophic marine bacteria. A genetic requirement for *galE* is evident for each of the above capabilities, expanding the known breadth of requirement for this gene in biofilm-related processes.

**IMPORTANCE:**

Heterotrophic marine bacteria have a central role in the global carbon cycle. Well-known for releasing CO2 by decomposition and respiration, they may also contribute to particulate organic matter (POM) aggregation, which can promote CO2 sequestration via the formation of marine snow. We find that two members of the prevalent particle-associated genus *Alteromonas* can form aggregates comprising cells alone or cells and chitin particles, indicating their ability to drive POM aggregation. In line with their multivalent aggregation capability, both strains produce TEP, an excreted polysaccharide central to POM aggregation in the ocean. We demonstrate a genetic requirement for *galE* in aggregation and large TEP formation, building our mechanistic understanding of these aggregative capabilities. These findings point toward a role for heterotrophic bacteria in POM aggregation in the ocean and support broader efforts to understand bacterial controls on the global carbon cycle based on microbial activities, community structure, and meta-omic profiling.

## INTRODUCTION

Marine bacteria are primary drivers of nutrient cycling in marine ecosystems, with an increasingly recognized role in the decomposition of particulate organic matter (POM) such as deceased phytoplankton cells and other detritus (1). POM-degrading bacteria exert their effects by the production of extracellular hydrolytic enzymes, releasing dissolved organic matter (DOM), some of which is consumed by the proximate bacteria and some of which diffuses away (2–4). Particle attachment and aggregate formation appear to be common bacterial behaviors related to POM degradation; this makes sense in light of the physical challenge bacteria face to uptake dissolved nutrients coming from the particle surface before they are lost to diffusion (5–8). Moreover, since individual bacteria may only rarely encounter particle hot spots, sticking to particles can provide extended access to high nutrients, supporting greater population growth (9).

Certain taxonomic groups of marine bacteria, including gammaproteobacteria in the genera *Vibrio* and *Alteromonas*, are frequently found enriched in POM-associated communities by metagenomics and molecular barcode studies (10–12). Similarly, *Vibrio* and *Alteromonas* are highly represented in particle-based enrichment cultures (3, 13). Members of the family *Alteromonadaceae* rapidly increase in abundance following high-molecular-weight DOM amendment as well, reflecting multifaceted abilities in organic matter utilization (14). Together, these findings indicate that members of these groups are adapted to attachment and surface-bound growth on POM. There is a growing interest in studying isolates of these genera in the laboratory to gain insight into their apparent specialization on POM in the ocean.

Isolates of *Vibrio* and *Alteromonas* have been cultivated in labs across the world, revealing details of the capabilities and mechanisms supporting their particle-associated lifestyle (8, 15–20). In discussing these, we consider several capabilities shared widely in bacteria (beyond *Vibrio* and *Alteromonas*): attachment to surfaces or particles (attachment), formation of surface-associated biofilms (biofilm formation), and formation of suspended aggregates (“aggregation,” sometimes called auto-aggregation, auto-agglutination, or flocculation). There has been comparatively more investigation in attachment and biofilm formation (for example, in *Vibrio cholerae* and *Pseudomonas aeruginosa*), whereas the study of aggregation is less well-developed (21–25).

Laboratory studies of attachment and biofilm formation in *Vibrio* spp. have revealed numerous genetic requirements. *V. cholerae* is among the best-characterized bacteria for surface attachment and biofilm formation, with established knowledge of the major components of its attachment machinery and biofilm matrix and many relevant signaling and regulatory pathways (23, 26–30). Particularly relevant to the particle-associated marine lifestyle, the molecular basis of chitin attachment and degradation has been detailed in *V. furnisii* (20, 31, 32). Other biofilm-forming *Vibrio* species such as *V. parahaemolyticus*, *V. vulnificus*, and *V. harveyi* have also been characterized. While they share similar genetic requirements for attachment and biofilm formation, they differ in their regulation of these capabilities (33). The wealth of knowledge from *V. cholerae* has provided a valuable point of reference to those studying attachment, biofilm formation, and aggregation in environmental *Vibrio* isolates (34).

The biofilm-forming *V. cholerae* and *P. aeruginosa* are also capable of forming suspended aggregates in liquid culture and in the human body, and these aggregates share some of the same properties as surface biofilms (35–38). Although they share the ability to aggregate in liquid culture, these two species differ in the structures required [extracellular polysaccharide (EPS) vs proteinaceous adhesins] and in the growth phase in which aggregation occurs in culture (during growth vs in the stationary phase) (36, 39–42). Given this diversity in the molecular requirements for aggregation in these well-studied species, it is crucial to characterize aggregation and its requirements in other genera. Moreover, while both *Vibrio* and *Alteromonas* can be found enriched on particles in coastal ecosystems, *Alteromonas* are more prevalent in the open ocean, highlighting the importance of investigating members of this genus (43).

So far, there are few studies on the molecular aspects of aggregation, attachment, or biofilm formation in *Alteromonas* spp. *Alteromonas macleodii*, the type species of the genus, is an emerging model species for laboratory study of particle- and phytoplankton-associated marine bacteria, with prior work across various strains examining alginate particle attachment, metabolic interactions with phytoplankton, and the core vs accessory structure of the pan-genome (12, 44–48). The *A. macleodii* type strain ATCC 27126^T^—first isolated from surface seawater near Hawaii—has recently become the subject of molecular investigation, revealing differential transporter expression under carbon vs iron limitation and the genes required for production of the siderophore Petrobactin (49–51). A recent study has reconstructed several metabolic pathways of this strain by manual curation of its genome annotation (52). We use this strain in this study and refer to it as “27126.” The other strain used in this study is the unclassified *Alteromonas* sp. ALT199 strain 4B03 (4B03), isolated from a chitin enrichment culture from Nahant, MA (13). Both strains exhibit aggregation in the lab, which we have sought to characterize and present below.

Here, we present two aggregation behaviors shared by 4B03 and 27126: They both aggregate during growth in Marine Broth-rich medium but grow planktonically in acetate minimal medium. Furthermore, both strains form aggregates with chitin particles when not growing. We identify a spontaneous non-clumping variant of 4B03 and determine its genetic polymorphisms by comparative genomics, then use reverse genetics in 27126 to demonstrate a genetic requirement for UDP-glucose-4-epimerase (encoded by *galE*) for wild-type aggregation capabilities. Lastly, we show that these *galE* mutant strains are deficient in producing large transparent exopolymer particles (TEP), suggesting that they produce a less sticky EPS than 4B03 or 27126. These findings provide an initial characterization of aggregation in *Alteromonas* spp., with potential implications for the particle-associated lifestyle of these bacteria in the ocean and value for inferring ecological function in meta-omics studies.

## RESULTS

### *Alteromonas* strains 4B03 and 27126 exhibit aggregation in rich medium

**Fig 1:**
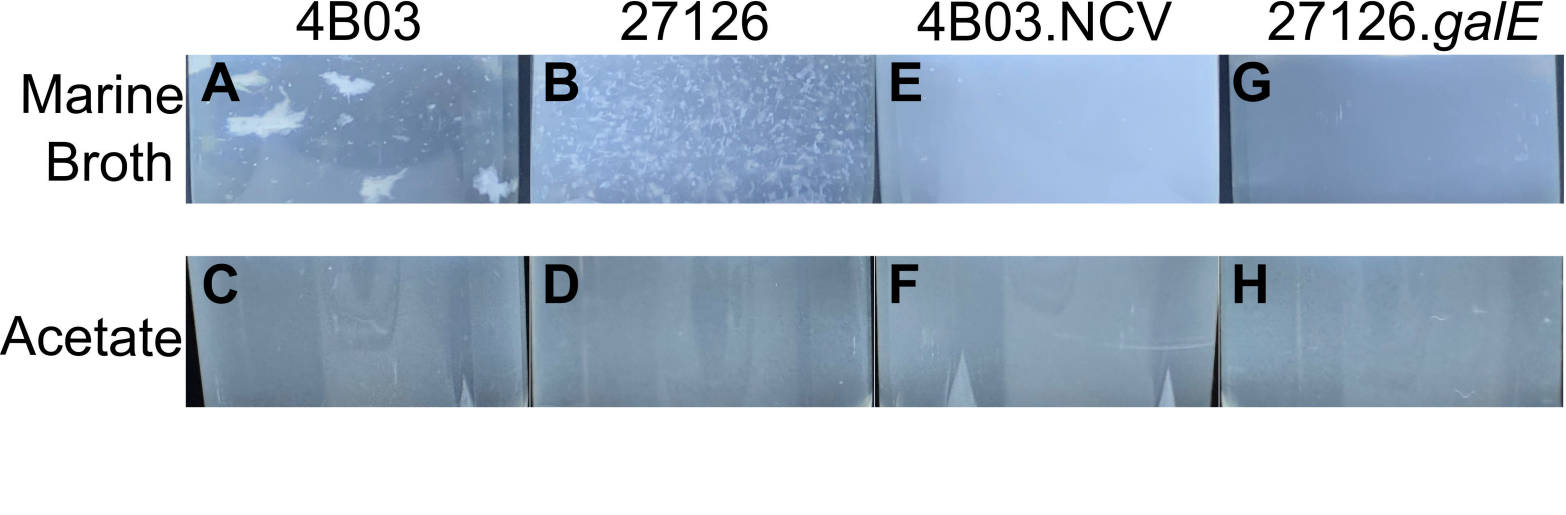
*Alteromonas* strains 4B03 and 27126 exhibit aggregation during growth in Marine Broth (MB) but grow planktonically in minimal medium with acetate (Ac) as sole organic nutrient. Photographs were taken after transfer to the specified media from saturated overnight Marine Broth cultures (2 h after transfer for MB tubes, 6 h for acetate). Tubes were illuminated from below by an LED light panel and then imaged from the side with a black background to better detect aggregates. Images are cropped to remove glare on the bottom of the tube and at the liquid-air interface. Some glare is still evident as whitish triangles on the bottom of the tube, and these are from the corners of the light panel. (**A**) 4B03 in Marine Broth, (**B**) 27126 in Marine Broth, (**C**) 4B03 in acetate, (**D**) 27126 in acetate, (**E**) spontaneous non-clumping variant of 4B03 (4B03.NCV) in Marine Broth, (**F**) 4B03.NCV in acetate, (**G**) *galE* knock-out 27126 Δ*galE::Km^r^* (27126.*galE*) in Marine Broth, (**H**) 27126.*galE* in acetate.

Strains 4B03 and 27126 form large aggregates visible to the eye when growing in Marine Broth (Difco 2216) (Fig. 1A and B). In contrast, these strains grow planktonically in minimal media with acetate as the sole organic nutrient (Fig. 1C and D). By planktonically, we specifically mean appearing to be fully suspended as single cells. To assess the extent to which Marine Broth elicits aggregation in these strains and verify that aggregates were not an artifact of inoculation from agar plates, we pre-culture 4B03 and 27126 in acetate overnight and then transfer planktonically growing cells to Marine Broth. We find that both strains form visible aggregates (>0.5 mm) within 1 hour post-transfer (Fig. S1), confirming that Marine Broth elicits rapid aggregation of initially planktonic cells.

A spontaneous phenotypic variant of 4B03, first identified in 2017 (see Materials and Methods), does not appear to aggregate in Marine Broth or acetate (Fig. 1E and F) and will be referred to henceforth as the “non-clumping variant” (NCV) or 4B03.NCV. 4B03.NCV has been used previously to examine the strain’s metabolic capabilities and interactions with chitopentaose-degrading *V. natriegens* (30).

### The non-clumping variant contains a 17-residue deletion in UDP-glucose 4-epimerase

**Fig 2:**
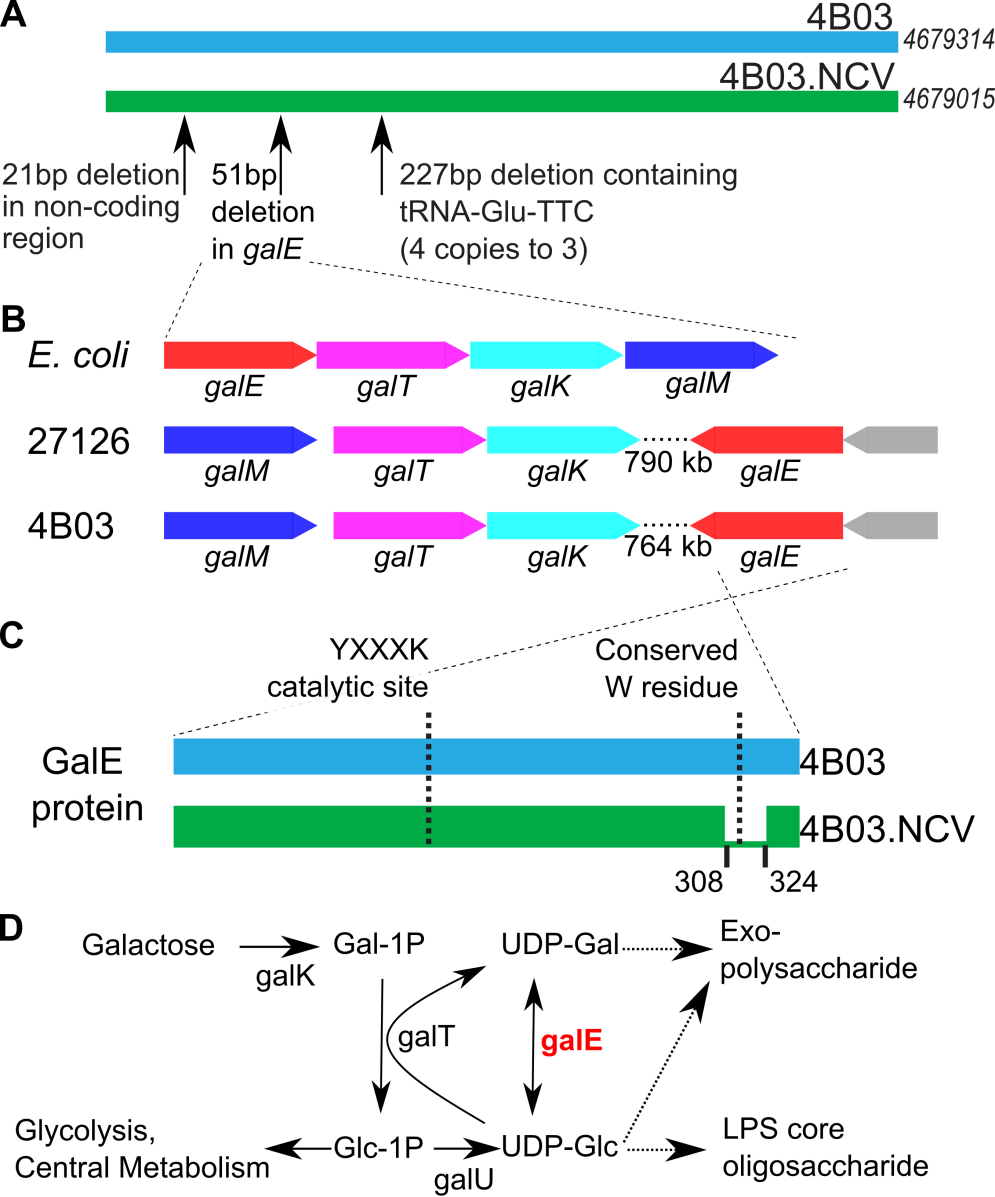
Genotyping the 4B03 non-clumping variant. (**A**) Schematic genome alignment showing three mutations identified in 4B03.NCV, genome lengths shown on the right; (**B**) operon structure of *galE* and related genes in 4B03 and 27126 compared to *E. coli* (gray gene in 27126 and 4B03: small hypothetical protein); (**C**) schematic protein alignment illustrating deletion of AAs 308–324 of GalE in 4B03.NCV. (**D**) Metabolic diagram showing reaction catalyzed by GalE and nearby products. Solid arrows represent singe-step enzymatic reactions, and dashed arrows represent multiple steps. Note that GalT reaction is reversible, but arrows are shown in one direction (the predicted direction when galactose is provided) for clarity. Adapted from Nesper et al. (55).

We performed whole-genome sequencing of 4B03 and 4B03.NCV to determine what mutations were present in the NCV. Genome comparison revealed a 21 bp deletion in a non-coding region, a 227 bp deletion containing one of the four copies of tRNA-Glu-TTC, and a 51 bp deletion within a gene predicted to encode UDP-glucose-4-epimerase (Biocyc Locus tag G1RG0-1423) (Fig. 2A). We refer to this gene henceforth as *galE* based on its similarity with the *galE* gene of *E. coli* (59% AA identity) (53). As there are no other significant BLAST hits to *galE* of *E. coli* in the 27126 or 4B03 genomes, we predict that *galE* has the same basic function in these strains as it does in *E. coli* (54).

In *E. coli*, *galE* is the first gene of an operon followed by *galT* (encoding UDP-transferase), *galK* (encoding galactokinase), and *galM* (encoding galactose mutarotase), with the latter two genes needed specifically for galactose utilization and the first two genes needed for cell wall synthesis regardless of galactose utilization, differentially regulated by the action of a small RNA (53, 56, 57). The *galE* genes of 27126 and of 4B03 are not found in the galactose utilization operon, which only includes *galM, galT,* and *galK* in these strains (Fig. 2B). Instead, *galE* is >750 kb away in its own operon with another gene upstream encoding a small hypothetical protein.

Alignment of *galE* from 4B03.NCV vs 4B03 reveals a 51 bp deletion near the C terminus of the protein (amino acids 308–324 in the sequence of 4B03; Fig. 2C). This deletion is not near the conserved catalytic site YXXXK at positions 150–154; however, it does include a highly conserved tryptophan at position 315 (Fig. 2C) (58–60). To assess whether the *galE* Δ308–324 mutation lead to loss of function in the GalE protein, we compared the growth capabilities of 4B03 and 4B03.NCV in acetate with or without added galactose (Fig. S2C and D). The two strains showed approximately the same growth rates in acetate alone, and the addition of galactose increased the growth rate of 4B03. However, the addition of galactose inhibited growth in 4B03.NCV, consistent with loss of function of the GalE protein, which can result in the accumulation of UDP-galactose (61, 62). Since *galE* Δ308–324 was the only mutation identified in a single-copy gene, we considered it the most likely to be responsible for the non-clumping phenotype.

### Disruption of galE in 27126 leads to loss of aggregation

In considering whether the *galE*Δ308–324 mutation could be responsible for the lack of aggregation in 4B03.NCV, we found that mutants of homologs of this gene have been associated with deficient biofilm formation in other bacteria, including *V. cholerae* and *Bacillus subtilis* (55, 62). The GalE protein catalyzes the conversion between UDP-glucose and UDP-galactose, monosaccharide derivatives that are used in synthesis of EPS and LPS (Fig. 2D) (55). Since EPS and LPS are important for aggregation and biofilm formation in other bacteria, we sought to make a targeted knockout in *galE* to test whether this gene is necessary for aggregation in *Alteromonas* spp.

While targeted gene disruptions have not been established in 4B03, 27126 has proven amenable to genetic manipulation (49, 63). Since 27126 forms aggregates when grown in Marine Broth similar to 4B03 (Fig. 1A and B), we sought to use this emerging model organism to test the necessity of *galE* for aggregation in *Alteromonas* spp.

A kanamycin resistance gene was inserted in the middle of *galE* in 27126 (Locus tag MASE_04285/MASE_RS04240) using homology-directed mutagenesis (Fig. S2A and B) (49), and the resulting mutant (27126 Δ*galE*::km^r^*,* referred to as 27126.*galE*) exhibits a loss of aggregation in Marine Broth (Fig. 1G). Resequencing 27126.*galE* confirmed the intended Δ*galE*::km^r^ disruption, with neighboring genes left intact (Fig. S2B). Since *galE* is the second gene in a 2-gene operon separate from the rest of the galactose utilization genes in 27126 (Fig. 2B), there is no apparent risk of polar effects from the Δ*galE*::km^r^ mutation in 27126.*galE*.

The loss of aggregation in 27126.*galE* compared to 27126 qualitatively matches the difference between 4B03.NCV and 4B03 (Fig. 1). Like 4B03.NCV, 27126.*galE* shows galactose sensitivity during growth in acetate + galactose (Fig. S2E and F), providing functional evidence for the loss of GalE protein activity. To make a clearer comparison of the aggregation capabilities among our strains, we then made use of several different methods to measure aggregate formation in batch culture, presented below.

### Quantifying aggregation by sedimenting fraction and by aggregate size distributions

#### Sedimenting fraction

**Fig 3:**
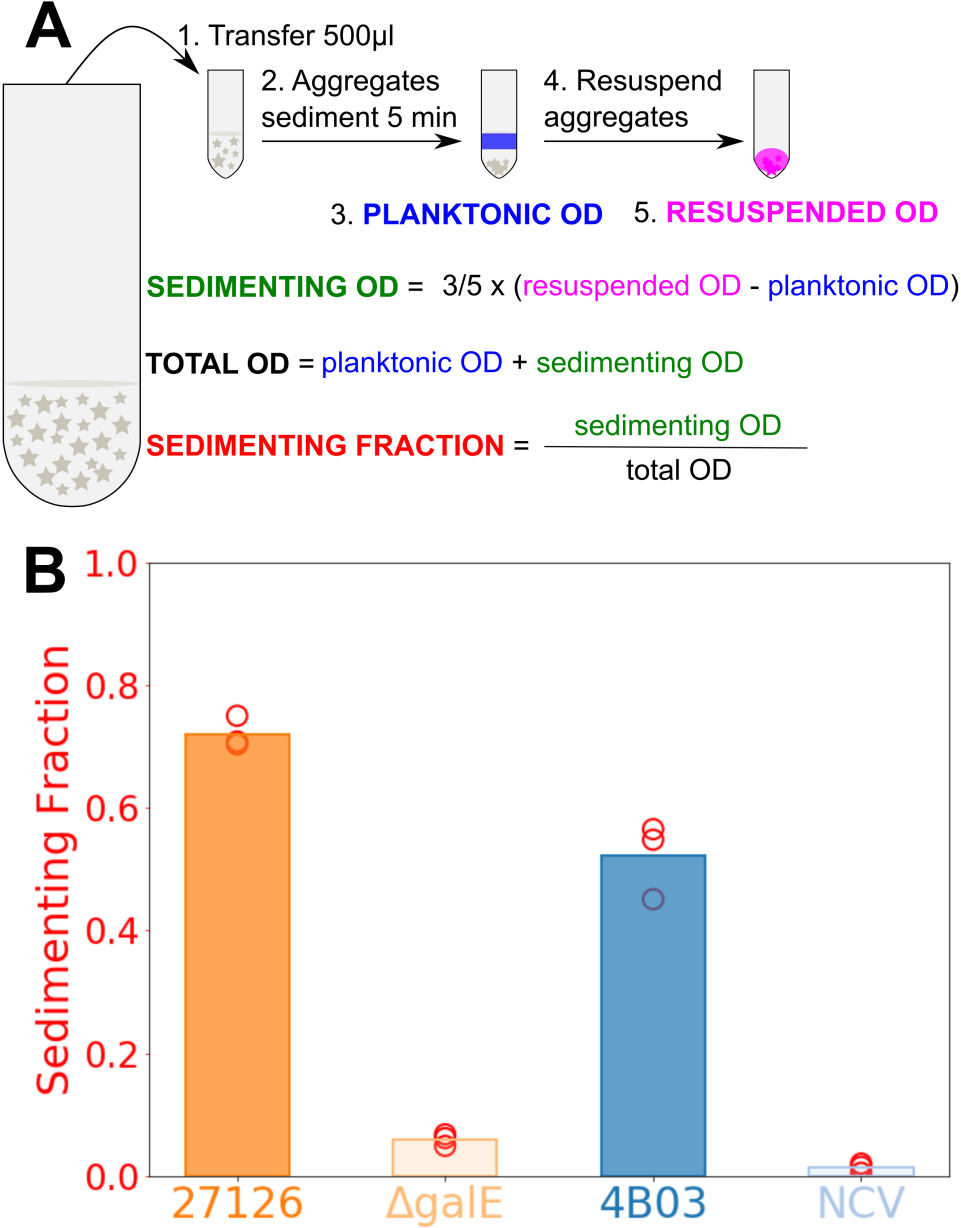
Measurement of aggregation by sedimenting fraction of OD. (**A**) To separate aggregates from bulk planktonic cells, 500 µL culture samples are removed and given 5 minutes for gravitational sedimentation. Sedimenting OD is 3/5 of the difference between resuspended OD and planktonic OD to account for the slight concentrating effect of letting aggregates from 500 µL sediment and then resuspending them in a smaller volume (300 µL). Sedimenting fraction is quantified as the portion of total OD that is in clumps of cells large enough to sink out of the top 200 µl within 5 min (see Materials and Methods: Measurement of aggregation by OD). (**B**) Comparison of aggregation as sedimenting fraction among strains 1 h after introduction to Marine Broth from acetate. Strain 27126.*galE* is abbreviated as “ΔgalE” and 4B03.NCV is abbreviated as “NCV.”

We employed an OD-based method to quantify the functional aggregation phenotype as defined by removal (sinking) of aggregated biomass in culture (Fig. 3A; Materials and Methods). This method was applied to cultures of 27126, 4B03, and their *galE* mutants growing in Marine Broth (Fig. S3), and the virtually complete loss of aggregation was evident in the difference in sedimenting fraction for 27126.*galE* compared to 27126, and similarly of 4B03.NCV compared to 4B03 (Fig. 3B). Thus, the qualitative differences in aggregation by visual assessment closely match the quantitative differences in aggregation by sedimenting fraction (Fig. 1A, B, E and G vs Fig. 3B). Moreover, this shows that the Δ*galE*::km^r^ mutation is sufficient to eliminate aggregation in 27126. These results strongly suggest that while there are several mutations present in 4B03.NCV, the *galE* Δ308–324 mutation alone is sufficient to account for the loss of aggregation in this strain.

#### Aggregate size distributions: Marine Broth

The mutants 4B03.NCV and 27126.*galE* did not form aggregates in Marine Broth according to visual inspection, and the functional defect in their ability to form sinking aggregates was shown by sedimenting fraction. Still, it is possible they formed microscopic aggregates too small to see by eye, with sedimenting speeds too slow for detection by the OD-based method. To assess this possibility, we used microscopy to analyze the occurrence of single cells vs aggregates during rapid aggregation in Marine Broth.

**Fig 4:**
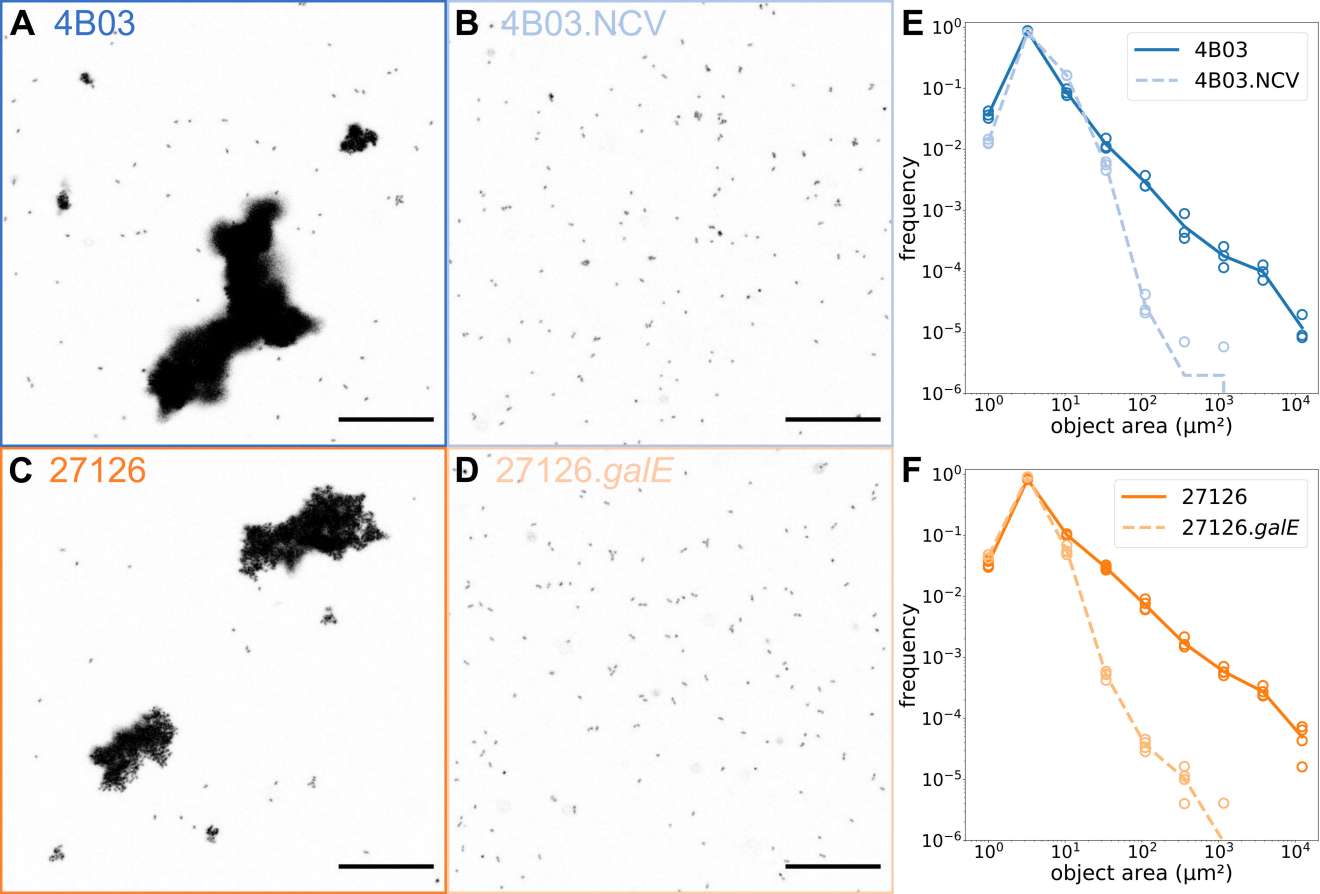
Microscopic evaluation shows differences in aggregation behavior at single-cell scale: (**A**) 4B03, (**B**) 4B03.NCV, (**C**) 27126, and (**D**) 27126.*galE*. Cultures were collected 30 min after transfer to Marine Broth, fixed with glutaraldehyde, and stained with SYTO 9. Scale bar = 50 µm. (**E and F**) Histograms (10 bins logarithmically spaced from 1 µm^2^ to 4 × 10^4^ µm^2^) of object sizes vs frequency collected by tile scan of a large field of view, see Materials and Methods. Points represent replicates and line represents mean. (**E**) 4B03 vs 4B03.NCV and (**F**) 27126 vs 27126.*galE*.

Toward this end, planktonic acetate precultures of 4B03, 4B03.NCV, 27126, and 27126.*galE* were transferred to Marine Broth at low density (initial OD range 0.026–0.037), incubated with shaking for 30 min, and then fixed and labeled with DNA stain SYTO 9 for microscopic analysis; see Materials and Methods. After only 30 min, cultures of 4B03 and 27126 had already formed aggregates reaching 50–100 μm in width as seen in micrographs (Fig. 4A and C). Mutant strains were present predominantly as single cells, with occasional small clusters of cells (Fig. 4B and D).

Figure 4E and F show the distribution of the areas of the cells and aggregates identified from image analysis (see Materials and Methods). All four cultures exhibit a clear peak near 3 µm^2^, which we identify as the single-cell peak given the characteristic 1 µm × 2–3 μm dimensions of *A. macleodii* cells (64). The solid lines show the frequencies of objects of each size for 4B03 (blue) and 27126 (orange), with maximal object areas of ~10^4^ µm^2^. Assuming the aggregates to have spherical shapes (to estimate volume from area), this would correspond to maximal aggregate volumes of ~10^6^ µm^2^ for 4B03 and 27126, or clusters of ~10^5^ cells for the two strains (assuming complete filling, given single-cell volume of a few μm^3^). These estimations are given for reference although aggregate parameters such as fractal dimension, porosity, or shape would be important for rigorous volume calculation. On the other hand, the maximum aggregate areas detected for 4B03.NCV or 27126.*galE* were ~10^3^ µm^2^, and the frequency at this size was 1–2 orders of magnitude below that of their respective ancestors 4B03 or 27126 (dashed lines vs solid lines, Fig. 4E and F). These data confirm that mutant strains 4B03.NCV and 27126.*galE* with respective mutations *galE* Δ308–324 and Δ*galE*::km^r^ have nearly completely lost the wild-type ability to aggregate.

#### Aggregate size distributions: chitin

**Fig 5:**
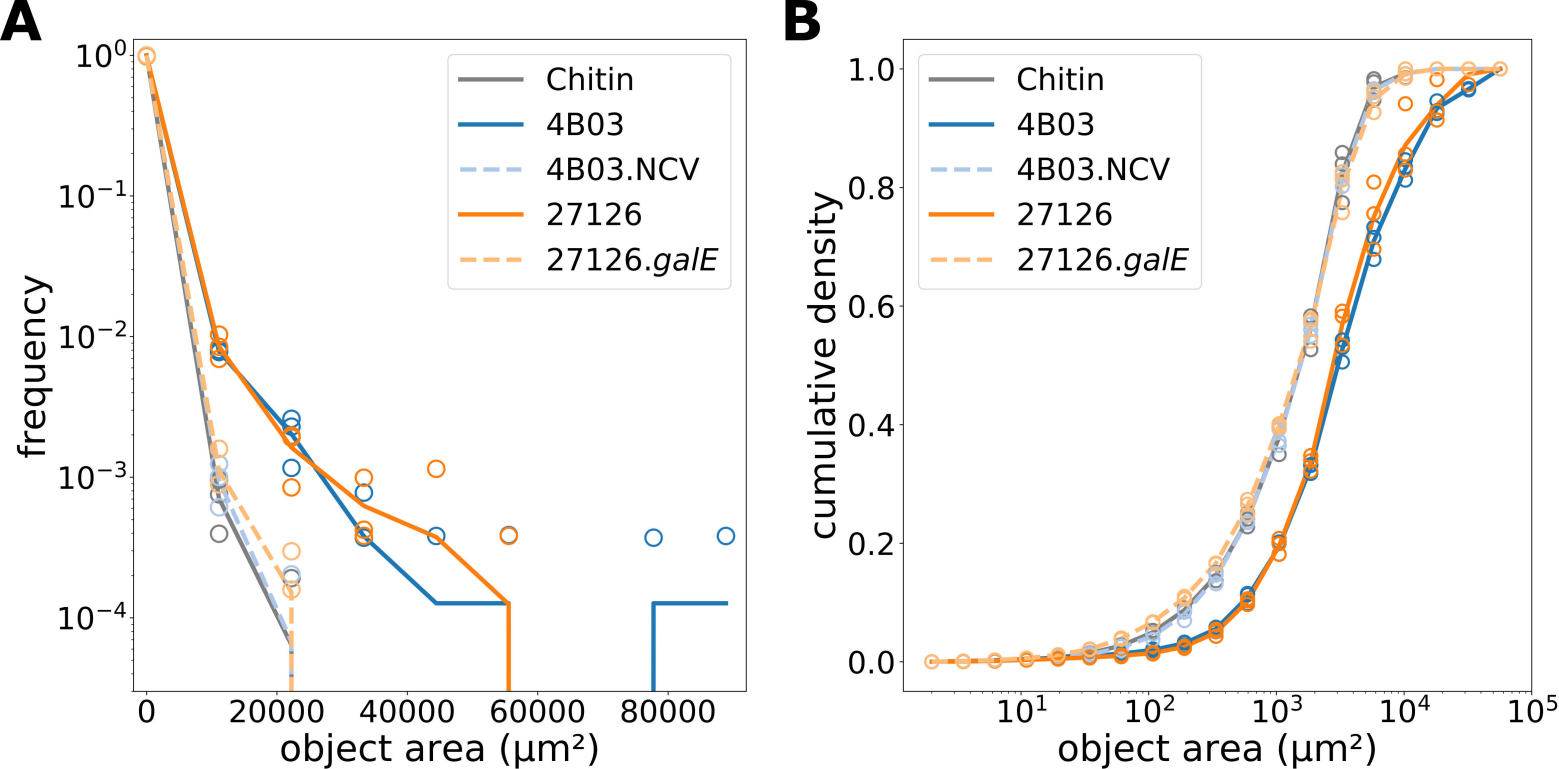
Size distribution of chitin particles (0.1% suspension) alone or with the addition of 4B03, 4B03.NCV, 27126, or 27126.galE after shaking 24 h. Chitin particles were specifically labeled with WGA-fluorescein. The histogram in (**A**) was created by counting objects in 10 linearly spaced bins from 2 to 10^5^ µm^2^ and then converting to frequency by dividing the count in each bin by the total. The histogram in (**B**) was created by counting objects in 20 logarithmically spaced bins from 2 to 10^5^µm^2^, then plotting cumulative sum of area at each bin divided by total area to show cumulative density.

Strains 4B03 and 27126 are also able to aggregate chitin particles. Unlike Marine Broth aggregation, this behavior is observed in the absence of growth: precultures growing exponentially in acetate minimal medium were transferred to minimal medium whose sole sources of carbon and nitrogen were chitin particles, which these *Alteromonas* strains cannot growth on (64, 65). While 27126 is able to grow on the constituent monomer of chitin (*N*-acetyl glucosamine or GlcNAc), this capability is variable in *Alteromonas* spp. and absent in 4B03 (64, 65). Aggregation with chitin particles is observed over a longer timescale than aggregation in Marine Broth (24 h vs 1 h). We compare the ability of the different strains to aggregate with chitin particles in Fig. 5.

The ability of wild-type strains to clump together multiple chitin particles is evident when examining the size distribution of chitin (chitin particles labeled with WGA-fluorescein lectin) and how it is affected by the addition of each strain during an overnight incubation. By collecting a large field of view and measuring the size of many chitin-containing objects (particles/aggregates), we can see that the chitin size distribution shifts upward with the addition of 4B03 and 27126, but not with the addition of 4B03.NCV or 27126.*galE* (Fig. 5). The suspension of chitin particles alone had a maximum object size of approximately 2 × 10^4^ µm^2^ (solid grey line, Fig. 5A), reflecting the fact the particles were passed through a 53-µm sieve before addition (see Materials and Methods). The addition of 4B03 or 27126 led to the formation of chitin-containing aggregates exceeding 4 × 10^4^ µm^2^, and some larger than 8 × 10^4^ µm^2^ (solid blue and orange lines, Fig. 5A). In contrast, addition of 4B03.NCV or 27126.*galE* led to no discernable increase in the size of chitin particles (dashed lines, Fig. 5A). The ability of 4B03 and 27126 to increase the size distribution of chitin particles was also evident in a cumulative density plot, in which 4B03.NCV and 27126.*galE* addition closely resembled chitin alone, but 4B03 and 27126 led to a distinct upward shift in the distribution of object area (Fig. 5B).

Cells and chitin particles were also imaged in 3D using confocal microscopy to reveal the arrangement of cells on and among the irregularly shaped chitin particles (Fig. S4). 4B03 and 27126 aggregate with chitin particles, forming clusters of cells on the particle surface that often seem to bridge or adhere two particles together (Fig. S4A and C). In contrast, mutant strains do not aggregate with chitin particles (Fig. S4B and D). These images give a qualitative view of how 4B03 and 27126 promote aggregation of chitin particles: sticky aggregates of cells may essentially trap or collect chitin particles, bringing multiple together.

### 4B03.NCV and 27126.galE are deficient in producing large Transparent Exopolymer Particles

Because the mutant strains are deficient in cell-cell and cell-particle aggregation, it suggests that they lack the ability to produce a substance with a general pro-aggregative effect. Extracellular Polymeric Substances (EPS) of this type are commonly studied in biofilm research, and in marine research, there is a focus on Alcian Blue-stainable EPS known as Transparent Exopolymer Particles (TEP). TEP are operationally defined by their ability to (a) be retained on filters with pores 0.4 µm or larger, and (b) bind the stain Alcian Blue, which is specific to acidic polysaccharides (66). Since GalE interconverts UDP-glucose and UDP-galactose, both of which may be substrates for synthesis of extracellular glycans, we hypothesized that *galE* mutants may be deficient in production of extracellular glycans such as EPS or TEP.

**Fig 6:**
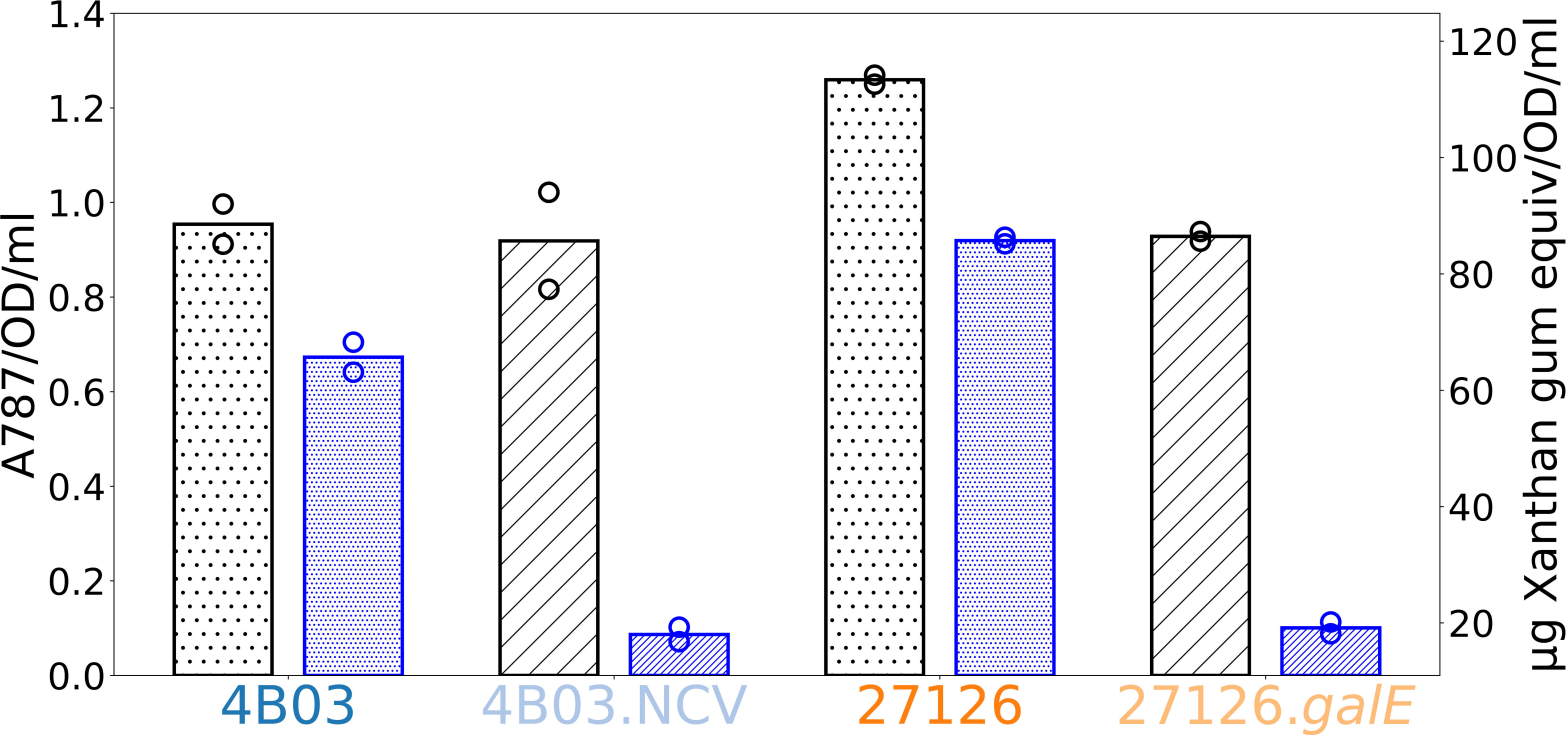
Size-specific TEP measurements for each strain 1 h after transfer to Marine Broth. Wild type strains 4B03 and 27126 are shown with dot fill, while strains 4B03.NCV and 27126.*galE* with mutations in *galE* are shown in diagonal stripe fill. One milliliter of culture (OD < 0.1) was filtered at 0.4 µm (light fill, black) or 10 µm (heavy fill, blue) pore size under low vacuum, and retained material was stained with Alcian Blue and rinsed with milliQ water. Bound dye was eluted with 80% sulfuric acid and absorbance was measured at 787 nm, with filtered media blanks subtracted for correction. Absorbance values were normalized to cell density by total OD (see Materials and Methods). On the right axis, TEP concentrations are given as microgram xanthan gum equivalents, estimated by a standard curve as described in Materials and Methods and shown in Fig. S5.

TEP production in strains 4B03, 4B03.NCV, 27126, and 27126.*galE* was determined 1 h after transfer to Marine Broth from acetate preculture (Fig. 6). To differentiate between TEP forming large particles and total TEP (which includes TEP associated with cells or forming small particles), samples were collected on 10 μm- and 0.4 μm-pore filters. Retained material was stained with Alcian Blue and rinsed, and then bound stain was eluted with sulfuric acid and measured by absorbance at 787 nm (A787). TEP values are reported both as A787 and as Xanthan Gum equivalents based on a standard curve (Fig. S5). The use of a Xanthan Gum standard curve in TEP measurements is widely encouraged to address variability in the staining activity of different preparations of Alcian Blue (66, 67).

Strains 4B03 and 4B03.NCV were found to produce comparable amounts of total TEP > 0.4 µm (Fig. 6). Strain 27126 produced slightly more total TEP > 0.4 µm than 27126.*galE*. However, a clear difference was observed between wild-type strains and *galE* mutants in the production of TEP > 10 µm. We found that 4B03 and 27126 produced significant amounts of large (>10 µm) TEP in Marine Broth (heavy dot bars), while mutants did not (heavy striped bars). The large TEP measured in WT strains amounted to a majority (>60%) of the total TEP collected for these strains (heavy dot fill bars vs light dot fill bars), while in mutant strains, large TEP was a small minority of the total (~10%; heavy stripe bars vs light stripe bars).

Because 4B03.NCV and 27126.*galE* were deficient in their ability to form large TEP despite having comparable amounts of total TEP to their wild-type counterparts, it appears that the TEP produced in mutants with disrupted *galE* function is less conducive to large particle formation. While the exact manner in which TEP supports aggregation and particle formation in 4B03 and 27126 strains is not yet known, the finding that the TEP produced by mutants 4B03.NCV and 27126.*galE* is less conducive to large particle formation suggests that it is less sticky. Here, “sticky” is meant in a general sense and could refer to the ability of TEP to form gel particles with itself or could refer to the strength of interaction between TEP and the bacterial cell surface.

### Oceanographic prevalence of *galE* and signature of *Alteromonas*-like *galE* operon

**Fig 7:**
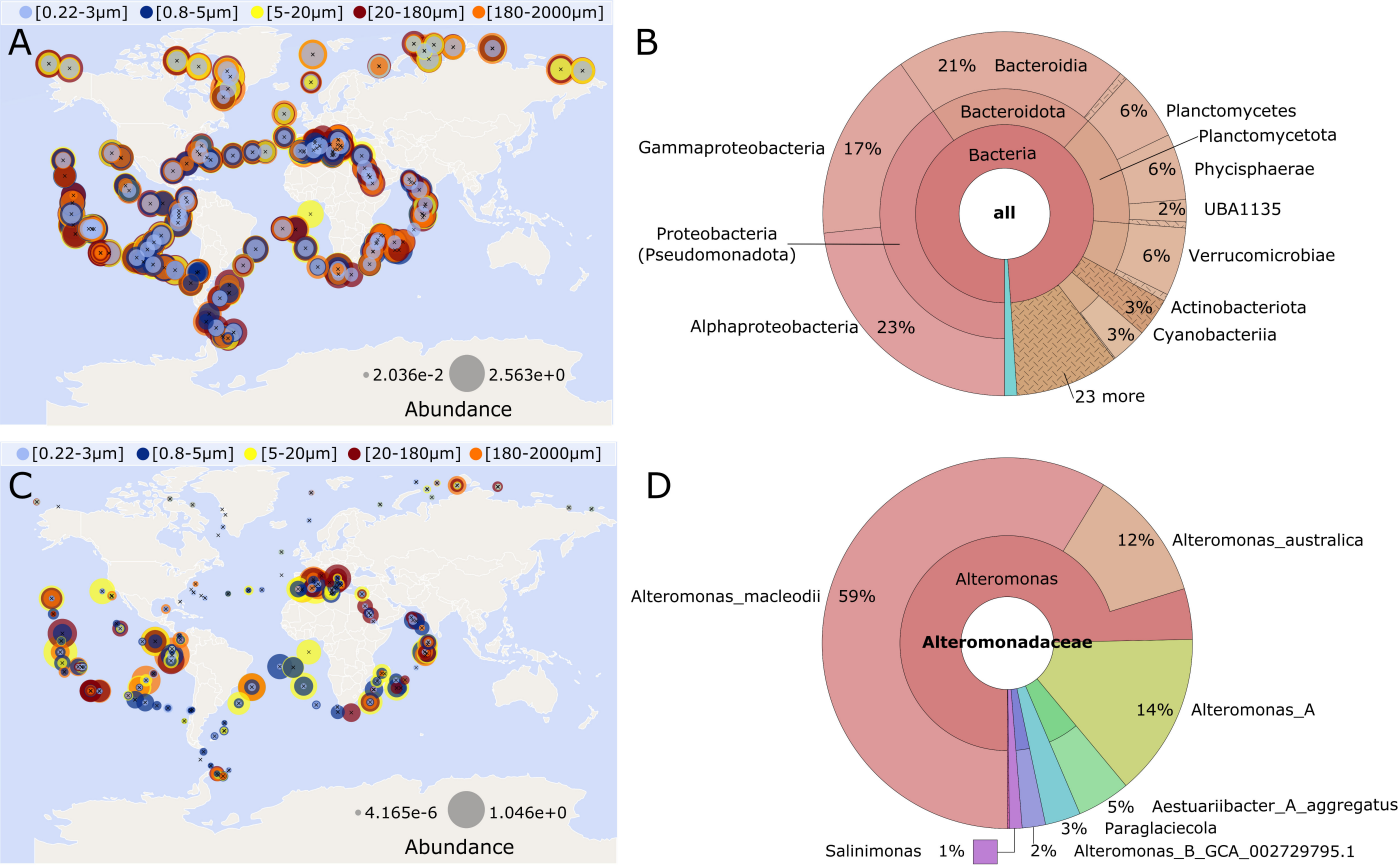
Tara Ocean Gene Atlas maps prevalence and taxonomic distribution of *galE* and *Alteromonas*-specific *galE*-associated hypothetical protein in surface metagenomes across ocean regions and particle size fractions. (**A and B**) blastp: 27126 UDP-glucose-4-epimerase (MASE_RS04240), database: BacArcMag; (**A**) global ocean abundance across size fractions; (**B**) taxonomic distribution of hits. (**C and D**) blastp: 27126 DUF6170 family protein (MASE_RS04245), database: BacArcMag; (**C**) global ocean abundance across size fractions; (**D**) taxonomic distribution of hits. Abundance values in A and C represent percent abundance of MAGs containing hit genes in metagenomes of each size fraction.

We used the Tara oceans online Ocean Gene Atlas to assess the prevalence of *galE*-like genes in metagenomes across ocean regions and size fractions (68, 69). When the amino acid sequence for *galE* from 27126 (MASE_RS04240) was used as a query, hits were detected at high abundance in all ocean regions and size fractions (Fig. 7A). However, the taxonomic distribution of homologs showed that these hits came from metagenomes across the bacterial phyla Proteobacteria (Pseudomonadota), Bacteroidota, Planctomycetota, and others (Fig. 7B). Thus, the prevalence of *galE* shown in Fig. 7A reflects the conservation of this gene among several of the bacterial phyla abundant in marine metagenomes, yet we sought to compare this to the oceanographic prevalence and size fraction distribution of *galE* in *Alteromonas* spp.*,* specifically.

To identify *galE*-associated genomic features that were unique to *Alteromonas*, we surveyed the genomes of 38 strains within *Gammaproteobacteria* for the number of UDP-glucose-4-epimerase genes, whether they were found in an operon, and what other genes were in the operon. We found that most strains had only one copy of *galE* in their genome, with the operon setting varying from family to family and in some cases among species of the same family (Table S1). The “*E. coli*-type” operon structure including *galT, galK,* and *galM* (Fig. 2B) was conserved among all surveyed members of *Enterobacteriaceae* and *Aeromonadaceae* and found in some members of *Vibrionaceae* and *Psychromonadaceae*.

The “*Alteromonas*-type” operon structure, with only a small hypothetical protein upstream (Fig. 2B; Fig. S2B), was conserved among all surveyed members of *Alteromonadaceae* but absent in genomes outside this family (Table S1). Moreover, this small hypothetical protein (MASE_RS04245, “DUF6170 family protein” in 27126) is the only copy encoded in the genome and generates no significant similarities in NCBI BLASTn when excluding *Alteromonodaceae* (taxid:72275). Although its function is unknown, we took this gene as an *Alteromonas*-specific *galE*-associated genomic feature and used it to query the Ocean Gene Atlas (Fig. 7C). Hits were found in surface metagenomes from across the temperate and tropical oceans, in all size fractions. All hits were from MAGs within the *Alteromonadaceae*, with 59% classified as *A. macleodii* (Fig. 7D). Most locations showed a higher abundance of hits in larger size fractions, 5–2,000 μm (Fig. 7C, largest circles are typically yellow, maroon, or orange), vs a low abundance in the smallest size fraction, 0.22–3 μm (Fig. 7C, light blue circles are usually much smaller). This trend is distinct from the broader distribution of metagenomes encoding *galE* shown in Fig. 7A, in which the abundance within the smallest size fraction is much more comparable to that in the larger size fraction. This distinction is indicative that among the many *galE*-encoding marine bacteria, *Alteromonas*, especially those that encode the *Alteromonas*-specific *galE*-associated hypothetical protein, are ecologically specialized to grow in aggregates and on particles.

## DISCUSSION

This is the first report within *Alteromonas* of the following capabilities, shared by strains 27126 and 4B03: (a) able to rapidly form macroscopic aggregates in Marine Broth, (b) able to aggregate chitin particles, and (c) able to produce sticky TEP. These capabilities expand the known phenotypic repertoire of these strains, which are emerging models for laboratory study of POM-associated bacteria. We consider all three of these capabilities potentially relevant to these strains’ particle-associated lifestyle *in situ*. Furthermore, this is the first report of a specific genetic requirement for capabilities of this type in *Alteromonas* spp. We will first discuss the implications of this genetic requirement and then expand to consider the potential impacts of these strains’ aggregation and TEP production capabilities.

The strains in this study share their requirement for *galE* in aggregation or biofilm formation with several other bacteria, including *V. cholerae, B. subtilis, Porphyromonas gingivalis, Xanthomonas campestris, and Thermus thermophilus* (55, 59, 62, 70, 71). Our finding that the *galE* gene is required for aggregation in *Alteromonas* spp. expands the known breadth of this requirement, which may be widely conserved among aggregative and biofilm-forming bacteria. In this case, *galE* may represent an effective target for biotechnological and medical efforts to control biofilm formation (58).

A recent pan-genome analysis of 12 isolates from *A. macleodii* has revealed roughly 3,000 core genes, 1,600 accessory genes shared among several strains, and 1,600 more unique genes found in only one strain (47). *galE* is included in the core genome of *A. macleodii*, suggesting that its requirement for aggregation and production of sticky TEP would likely apply across strains. Moreover, our comparison of *galE* operon context across the *Alteromonadaceae* and related families showed that the “*Alteromonas*-type” operon, with a small hypothetical protein upstream, was conserved among sequenced representatives of this family but not found in other families (Table S1). Some *Alteromonas* species have previously been described producing acid polysaccharides, some of which included galactose and galacturonic acid (64, 72, 73). However, these reports did not describe an effect of the EPS in aggregation or TEP formation.

Since UDP-glucose/galactose may also be substrates for the production of lipopolysaccharide (LPS), we considered whether the mutations in *galE* may affect LPS production. However, the structure of LPS has been determined in 27126^T^, and it was found to lack an O-antigen polysaccharide, consisting only of lipid A and the core oligosaccharide (74). The core oligosaccharide lacked any galactose-derived residues, being composed of Heparin, Kdo, and glucosamine residues. Since the LPS of 27126 does not appear to have a use for UDP-galactose, we consider it unlikely that the *galE* mutants studied here had deficient LPS formation although this could be determined by future experimental verification. Notably, strain variation in genomic regions annotated for LPS and EPS production has been found in the congeneric *A. mediterranea* (formerly known as *A. macleodii* “Deep Ecotype”) (75–77).

The rapid aggregation of strains 4B03 and 27126 in Marine Broth following planktonic growth in acetate has not previously been described in *Alteromonas* but may provide clues about their accumulation in particle-associated communities. 4B03 and 27126 can go from planktonic cells to aggregates 50–100 μm in length within 30 min (Fig. 4). These aggregates must be forming by collision and adhesion of initially planktonic cells, rather than by growth with retention of daughter cells since these strains only achieve 2–3× growth during the first hour (Fig. S3). Admittedly, these values for aggregate formation are not directly translatable to *in situ* marine conditions since the higher cell densities and shaking incubation in our experiments would be expected to speed up aggregate formation by increasing encounter rates (78). Still, the ability to rapidly initiate aggregation in 4B03 and 27126 may be advantageous in the context of growth on POM since particle encounters may be rare and fleeting for planktonic marine bacteria. Since the peptone and yeast extract in Marine Broth may resemble chemical signatures of cell lysis and POM hydrolysis, we speculate that the rapid aggregation of these *Alteromonas* spp. in Marine Broth may reflect their strategy for colonizing particles and help explain their enrichment in particulate communities *in situ*. In a study on marine bacteria isolated from enrichment cultures of diatom aggregates, Bidle and Azam found that one of these strains (Tw3, formerly classified as *Alteromonadaceae*, but now classified as *Psychrosphaera* within *Pseudoalteromonadaceae*) exhibited intense aggregation in Marine Broth as well, supporting a connection between this laboratory phenotype and the oceanographically-important process of diatom aggregate formation/colonization (3).

Examples of aggregation in rich medium have been reported across bacteria from different environments, and in some cases, a requirement for EPS production has been shown. In opportunistic human pathogen *P. aeruginosa*, aggregation is observed during growth in LB, with a dependence specifically on the *Psl* polysaccharide, but not *Pel* (36). In the legume root nodule-colonizing *Sinorhizobium meliloti*, aggregation is observed in TY-rich medium, with a dependence on *EPS II* galactoglucan (79). In human commensal *Mycobacterium smegmatus*, aggregation seems to be favored during growth in rich medium or glycerol, while pyruvate favors planktonic growth (80). In the marine-dwelling human pathogen *Vibrio fluvialis*, biofilm formation occurs during stationary phase in BHI-rich medium but is not detected in minimal medium (81). While what we have shown partially mirrors these previous studies, it extends the aggregation behavior to the oceanographically relevant genus *Alteromonas* and suggests new ecologically relevant functions, as discussed below.

In contrast to the rapid cell-cell aggregation and simultaneous fast growth that 4B03 and 27126 exhibit in Marine Broth, they are also able to form aggregates with chitin particles during overnight incubation in the absence of growth. In 4B03, this capability reflects its isolation as part of a chitin enrichment culture, where it is thought to have been a cross-feeder or scavenger, consuming byproducts and exudates of primary degraders (13, 18, 65). Since neither 4B03 nor 27126 can grow on chitin, their ability to aggregate with chitin particles in the absence of utilizable nutrients appears to be maintained during starvation. While the abilities and activities of bacteria during starvation are not well understood, prior studies in marine bacteria from *Alteromonas* and *Vibrio* have indicated an ability to maintain viability for days to weeks (82–84). Chitin aggregation during starvation in 4B03 and 27126 may be a conserved strategy for cross-feeding of metabolites from chitin degraders through aggregation of particles to create larger hotspots of DOC availability. 4B03 cannot grow on GlcNAc, the constituent monomer of chitin, suggesting that this strain may fill a “scavenger” role, consuming exudates and waste products of chitin degraders (19, 65). However, 27126 is able to consume GlcNAc, suggesting that this strain could be an “exploiter” benefiting from chitin degraders without contributing enzymes to chitin hydrolysis (19, 64). Alternatively, the ability to stick to chitin in 4B03 and 27126 may serve another purpose, such as attachment to chitinaceous diatoms or copepods.

Large aggregates of POM and bacteria that form in the upper ocean are known as marine snow, and their sinking exports organic matter from the upper water column to depth, sequestering C from exchange with the atmosphere (85, 86). TEP appear to be a major determinant of aggregation and marine snow formation, creating gel particles that can stick to phytoplankton, bacteria, minerals, and debris (87, 88). The finding that *Alteromonas* strains 4B03 and 27126 can produce TEP with sufficient stickiness to rapidly form large, sedimenting particles suggests that the aggregation behavior presented in this study may have relevance to TEP and marine snow formation in natural conditions. While there has historically been a focus on phytoplankton as the primary producers of TEP, it has been known for some time that heterotrophic marine bacteria can also produce significant amounts of TEP (89, 90). TEP production by bacteria has been found to vary with nutrient availability in a seawater microcosm enrichment study (91). The production of large TEP by *Alteromonas* spp. in test tubes indicates their potential contribution to this process *in situ*.

The deficiency of large TEP production in the mutant strains suggests that their aggregation defects are due to lack of stickiness in the EPS they produce and conversely that production of sticky TEP by wild-type strains 4B03 and 27126 enables their cell-cell and cell-particle aggregation capabilities. In phytoplankton, where TEP production has been studied most thoroughly, it has been found that species differ not only in TEP production, but also in the stickiness of TEP produced (92). Thus, there is precedent for variations in TEP stickiness, and it is possible that further study of the differences in EPS composition between WT and *galE* mutant strains of *Alteromonas* spp. could reveal the biochemical basis for differences in TEP stickiness and ability to form large particles.

## MATERIALS AND METHODS

### Strains and culture techniques

Strain 27126^T^ used in this study (NCBI BioSample ID SAMN02603229) is the type strain for the species *A. macleodii* (51, 93). It produces the siderophore petrobactin, and transcriptomic studies have revealed different carbon- and iron-specific deployment of TonB-dependent transporters (49, 50). Other strains of *A. macleodii* have been studied for their association with cyanobacteria *Prochlorococcus* and *Trichodesmium* (48, 94), for their ability to degrade aromatic hydrocarbons, or for their ability to hydrolyze and consume algal polysaccharides (45, 47, 95). We obtained strain 27126^T^ (referred to as “27126”) from DSMZ (DSM no 6062, ATCC 27126). The 27126 Δ*galE*::km^r^ insertion mutant (referred to as “27126.*galE*”) was generated from 27126 as described below.

Strain 4B03 is a representative of the unclassified species *Alteromonas* sp. ALT199 (NCBI Taxonomy ID 1298865), whose first isolate, “AltSIO,” was collected at the Scripps Institute for Oceanography in southern California (96). AltSIO was capable of consuming as much of the ambient dissolved organic carbon pool as complete natural assemblages and correspondingly exhibited a generalist capability to use many individual nutrients, suggesting the potential for a central role in C cycling (96, 97). The unofficial ALT199 species appears to be closely related to *A. macleodii* by multiple genomic comparisons (94, 98).

Strain 4B03 was isolated as part of a large isolate collection from chitin enrichment cultures of coastal surface bacteria in Nahant, MA (NCBI Biosample SAMN19351440) (13). It has been considered a “cross-feeder” in the context of chitin-degrading communities, as it does not grow on chitin or its monomer GlcNAc, but does grow on metabolic byproducts of the chitin degraders such as acetate (65). The non-clumping variant 4B03.NCV spontaneously arose in the process of maintaining and sharing the stocks of the WT strain among labs.

Strains were cultured by streaking out frozen glycerol stocks on Marine Broth (“MB,” Difco 2216) plates (1.5% agar). Colonies were grown overnight at 27°C or over two nights at room temperature. Plates were then stored at 4°C, and colonies were used to inoculate liquid media within 4 weeks of streaking. All liquid cultures were grown in a water bath shaker at 27°C and ~200 rpm. For all experiments (except Fig. 1), a two-step procedure for preparatory cultures was used to begin with cells in a reproducible physiological state. First, seed cultures were started by inoculating a single colony into 2 mL liquid MB and growing for 4–24 h. Then, precultures were prepared in Marine Biological Laboratories-inspired “MBL” minimal medium with 30 mM acetate as sole organic nutrient, 10 mM ammonium, 1 mM phosphate, 40 mM HEPES buffer, 4 mM Tricine, and trace metals including iron, but no vitamins provided (referred to as “acetate” throughout our study; full recipe in Amarnath et al.referred to as “strongly buffered” HEPES minimal medium) (99). Precultures were inoculated with cell suspensions prepared by centrifuging 1 mL of seed culture at 6,000 × *g* for 3 min, washing in 1 mL minimal medium, centrifuging again, and resuspending again in 1 mL minimal medium. Precultures were prepared in multiple dilutions and grown overnight so that cells could be collected from exponentially growing cultures at similar ODs the next day to start each experiment. This preculture approach allowed us to begin each experiment with cells in the same growth state (exponential growth in acetate minimal medium) and comparable densities across strains, enabling reproducibility and comparison among different strains and experiments.

### Photography of aggregation in culture tubes

For Fig. 1, saturated overnight Marine Broth cultures were centrifuged at 6,000 × *g* for 3 min, and cells were resuspended in fresh Marine Broth or acetate and inoculated 1:10 into the same media and then incubated until growth was evident (2 h after transfer for Marine Broth, 6 h after transfer for acetate). Then, tubes were removed from the shaker and dried with a paper towel before imaging. Images were collected on an iPhone 14 pro with default settings. Tubes were held over an LED light sheet to illuminate from below while imaging from the side, making it easier to detect aggregates. Tubes were swirled gently to suspend aggregates before capturing each image. Fig. S1 tube images were collected in the same manner, but before the start of the experiment, cells were precultured in acetate, collected in late exponential at OD 0.65–0.75, centrifuged and resuspended in either Marine Broth or acetate as above, and then diluted 1:20 in the medium in which they were resuspended.

### Genome sequencing and comparative genomics

Overnight cultures were prepared in Marine Broth for a single clone of *Alteromonas* 4B03 and 4B03.NCV. DNA was extracted and purified with the Promega Wizard genomic DNA purification kit. Genomes were sequenced by long-read (300Mbp) nanopore sequencing at the Microbial Genome Sequencing Center (now SeqCenter). Quality control and adapter trimming was performed with Porechop (v0.2.3_seqan2.1.1) (https://github.com/rrwick/Porechop). Assembly statistics were recorded with QUAST v5.0.2 (100). The genomes were annotated with the Rapid Annotation using Subsystem Technology tool kit (RASTtk) v2.0 with default settings for bacteria (101–103).

### Homology-directed disruption of *galE* gene

To generate a Δ*galE*::km^r^ mutation in 27126, we used conjugation to introduce the mobilizable plasmid pJREG1 (Fig. S2A), constructed using the Loop Assembly method (49, 104), containing a kanamycin resistance cassette flanked by two homology arms matching the 5′ and 3′ ends of the gene (Fig. S2B) into 27126 via an *E. coli* epi300 strain harboring the conjugative helper plasmid pTA-Mob (49, 105). Plasmid pJREG1 also contained a *SacB* gene conferring sensitivity to sucrose (Fig. S2A). Transconjugants were selected using kanamycin, and successful recombination of the KO cassette into the genome was selected by streaking onto sucrose + Km double selection plates. After re-streaking on the same double selection plates, a transconjugant colony was inoculated in Marine Broth, saved in a 25% glycerol stock, and designated 27126.*galE*.

Successful gene disruption was confirmed by resequencing 27126.*galE*. A single colony was inoculated in 10 mL Marine Broth and grown to OD ~1.25, and then 8 mL was pelleted by centrifugation and resuspended in 0.5 mL DNA/RNA shield (Zymo Research R1200). The resuspended cell pellet was then submitted to Plasmidsaurus for long-read nanopore sequencing. The genome assembly protocol involved trimming with Filtlong v0.2.1 (106) to eliminate low-quality reads, followed by downsampling the reads to 250 Mb via Filtlong to create an assembly sketch using Miniasm v0.3 (107). Based on the Miniasm results, the reads were downsampled to ~100× coverage, and a primary assembly was generated with Flye v2.9.1 (108) optimized for high quality ONT reads. Medaka (Oxford Nanopore Technologies Ltd.) was then employed to improve the assembly quality. Post-assembly analyses include gene annotation (Bakta v1.6.1), contig analysis (Bandage v0.8.1), and completeness and contamination estimation (CheckM v1.2.2) (109–111). The Δ*galE*::km^r^ mutation was confirmed by DNA alignment of the *galE* gene region between 27126 (using genome sequence GenBank CP003841.1) and 27126.*galE* in Benchling.

### Measurement of aggregation by sedimenting fraction of OD

Cultures containing a mixture of aggregates and planktonic cells were suspended by swirling, and then 500 µL was collected and transferred to a 2.0-mL microcentrifuge tube. After a 5 min sedimentation period, the top 200 µL was carefully removed and OD at 600 nm (OD) was measured, giving the planktonic OD. Then, aggregates in the bottom 300 µL were resuspended by vigorously pipetting up and down 10×, then 200 µL was removed to measure OD, giving the resuspended OD.

Sedimenting OD = 0.6× (resuspended OD − planktonic OD)

Total OD = planktonic OD + sedimenting OD

Sedimenting fraction = sedimenting OD/total OD

### Microscopy of cell clusters

Planktonic cultures of each strain grown acetate minimal medium were collected during exponential growth and 100 µL was inoculated directly into 3 mL pre-warmed MB (3–4 replicate tubes per strain). Initial density at inoculation was within a <2× range, from OD 0.026–0.037. All subsequent pipeting steps were performed gently with wide bore pipet tips (Thermo Scientific ART 2069G) to reduce physical disruption of aggregates. After 30 min, 200 µl of well-suspended culture was collected and fixed immediately by adding 800 µL glutaraldehyde 2.5% in 1× Sea Salts (“1xSS”: 342.25 mM NaCl, 14.75 mM MgCl_2_, 1.00 mM CaCl_2_, 6.75 mM KCl in milliQ water). After 15 min, fixed cells and aggregates were resuspended by gently inverting the tube, and 200 µL was transferred to 1 mL 1xSS containing 4 mM Tricine (pH 8.1) with 10 µM SYTO 9 (A DNA stain used to visualize the nucleoid of each individual cell; Invitrogen S34854) in a 4-chamber #1.5 coverglass assembly (Cellvis C4-1.5H-N; each chamber 9.3 mm × 19.9 mm), and allowed to settle overnight. SYTO 9 was chosen as a stain for its high contrast, general DNA-staining activity, and compatibility with fixed cells. Settled samples were imaged on a Leica SP8 confocal microscope with a 10× objective, zoom 4.0, 2× line average, and pinhole 5.0 to expand the optical section in Z (allowing detection of cells that were near but not quite at the bottom of the chamber). A large area was imaged by tile scan for 3–4 replicate chambers for each strain (one chamber for each replicate tube; 4B03,NCV[*n* = 3]: 94 mm^2^; 27126,ΔgalE[*n* = 4]: 118 mm^2^), with individual tiles automatically merged to a single image in the Leica Application Suite Advanced Fluorescence software (“LAS AF,” version 4.0.0.11706).

Image analysis was carried out in Python using the Sci-kit Image analysis package (112). Merged tilescan images were imported as TIFF, gaussian filtered to reduce noise, binarized to delineate objects, then object area in μm^2^ was measured using the stored pixel length information from image metadata. Tens of thousands of objects (cells and aggregates) were measured for each strain (4B03: [102,082-121,702], 4B03.NCV: [142,858-191,452], 27126: [62,213-69,757], 27126.*galE*: [245,238-300,727]).

### Microscopy of bacteria with chitin particles

Chitin size distributions were generated as follows. Planktonic cultures of each strain in acetate minimal medium were washed and resuspended in minimal medium without C or N source and then transferred at OD 0.06–0.07 to a 0.1% chitin suspension in the same minimal medium (5.5 mL final). The chitin particles used (Sigma C7170) were sieved to remove particles larger than 53 µm before being autoclaved in milliQ water as a 1% suspension. After 1 day shaking at 27°C in upright 25 mm borosilicate glass tubes, samples were prepared for imaging as follows: 400 µL of suspended cell + chitin mixture was gently transferred with a wide bore pipet tip to black microcentrifuge tubes containing 2 µL Syto60 (5 mM in DMSO, Invitrogen S11342), gently pipeted up and down once to mix, then fixed immediately by adding 800 µL glutaraldehyde 2.5% in 1xSS with a wide bore pipet tip and mixing by gently pipetting up and down once, then capping and gently inverting tube 2×. After 10 min, fixed samples were resuspended by gently inverting 2×, then 50 µL was carefully transferred with a wide bore pipet tip to three replicate wells containing 1,000 µL 1xSS with 25 µg WGA-fluorescein lectin to label chitin (Vector labs FL-1021) within a 4-chamber #1.5 cover glass (Cellvis C4-1.5H-N). Samples were imaged 20 h after loading microscopy chambers to allow chitin settling. Images were collected on a Leica SP8 confocal microscope using the Leica LAS AF software. Tile scans of approximately 5 mm × 10 mm were recorded, using a 10× objective, 4× zoom factor, and expanded pinhole of 5.0 Airy units to enable an optical section in Z of >50 µm. The fluorescein channel was analyzed to show the size distribution of WGA-labeled chitin particles. Image analysis was carried out in Python using the Sci-kit Image analysis package in the same manner described above (112).

The 3D Z-stack images of cells and chitin particles shown in Fig. S4 were generated as above, with the following specific modifications. Sieved chitin particles from the 53–106 µm size class were provided, and cultures were shaken for 7 days. Rather than collecting tile scans, Z-stack images were taken with a 40× NA 1.10 water immersion objective to show the organization of cells among particles. The Syto60 DNA dye intended to label bacterial cells was also taken up by chitin particles, but the WGA-Fluorescein lectin for chitin coated the surface of all particles. Laser power and gain settings were adjusted to enable differentiation of chitin particles based on WGA-Fluorescein despite high fluorescence of of chitin particles on the Syto60 channel used for detection of cells. 3D renderings were generated with the Leica LAS AF software, with adjustments to the intensity range of each channel made to optimize differentiation of cells and chitin particles.

### TEP determination

TEP was determined using an Alcian Blue dye-binding assay following Passow and Alldredge (66). A staining solution of 0.04% Alcian Blue (AB) in 0.6% acetic acid in milliQ water was prepared with a final pH of 2.55. The staining solution was 0.2 μm-filtered and kept at 4°C for <30 days. For each TEP measurement, 1 mL of culture at low OD600 (0.058–0.084) was filtered over polycarbonate filters with 0.4 µm or 10 µm pore size using low, constant vacuum pressure at ~200 mmHg. To dye retained TEP, 1 mL of AB staining solution was added to the filters, with constant pressure for 0.4 µm filters and with a < 1 min pause in vacuum for 10 µm filters (solution passes very quickly through 10 µm filters without pausing vacuum). After unbound staining solution was removed by vacuum, filters were rinsed with 1 mL milliQ water. Filters were carefully removed, the bottom side was dabbed on a Kimwipe to remove any adsorbed liquid, and then they were stored in glass scintillation vials. Bound Alcian blue was eluted from filters in 6 mL 80% sulfuric acid for 2–20 h with occasional agitation, and then absorbance at 787 nm was read using a Thermo Scientific Genesys 20 spectrophotometer. Absorbance was blanked with milliQ water, and a reference blank of 80% sulfuric acid was recorded. Filter blanks were prepared by repeating the staining procedure above with uninoculated media. Final A787 values were corrected by subtracting filter blank and 80% H2SO4 blank, and then they were normalized to OD600 measurements of cell density collected contemporaneously with culture filtration.

A standard curve for Alcian Blue labeling of acid polysaccharides was prepared with Xanthan Gum (“XG”; Sigma-Aldrich G1253, ordered 10/2022) using the updated method of Bittar et al. (67). A standard solution of 80 mg/L XG was prepared in 100 mL milliQ water (0.22 µm filtered) and gently swirled for 10 min until the material appeared to have completely dissolved. Then, dilutions were made with milliQ water to achieve 20, 40, and 60 µg/mL solutions at 1 mL final in 5 mL polypropylene snap-cap tubes. AB staining solution (500 µL) was added to each XG dilution and a tube containing 1 mL of pure milliQ water. Tubes were mixed by manual agitation for 1 min, leading to the formation blue stringy gel particles visible in the 60 and 80 µg/mL tubes. The entire tube contents were poured onto 0.4 μm-pore polycarbonate filters at low constant vacuum, and then filters and retentate were removed, gently dabbed on a Kimwipe to remove residual liquid on the bottom, and placed in scintillation vials. Alcian Blue was eluted with 6 mL 80% sulfuric acid for 2 h with gentle agitation and absorbance at 787 nm was read.

### *galE* operon comparison and oceanographic prevalence

To compare *galE* operon context in members of families near *Alteromonadaceae*, each strain’s database was brought up in BioCyc and searched for “epimerase.” The number of genes annotated “UDP-glucose-4-epimerase” was tabulated, and the operon context of each copy was assessed in genome browser (54).

To assess gene prevalence across ocean regions and particle size fraction, we used BLAST search of the Tara Ocean Gene Atlas (OGA), https://tara-oceans.mio.osupytheas.fr/ocean-gene-atlas/. Protein sequences were exported as FASTA from Biocyc and used to query the BAC-ARC-MAGS data set (Tara oceans bacterial and archael genomes) using blastp in the OGA (54, 68, 69). Maps of blast hit abundance and plots showing taxonomic distribution of homologs were exported as SVG and edited in Inkscape solely to increase text size and improve legibility.

## Data Availability

The *A. macleodii* ATCC 27126^T^ genome sequence used in this study was GenBank CP003841.1 (44). The genome sequences of *Alteromonas* sp. ALT199 strain 4B03 wild-type (BioSample Accession SAMN39273372), the non-clumping variant of 4B03 (SAMN39273373), and 27126 Δ*galE*::km^r^ (SAMN39273374) are available in NCBI GenBank (BioProject PRJNA1061545). Microscopy data and image analysis code are available on Zenodo (DOI: 10.5281/zenodo.11111667). All other data will be made fully available upon request.

## SUPPLEMENTAL MATERIAL

Supplemental material mbio.00038-24-s0001.pdf Fig. S1 to S5 and Table S1.

ASM does not own the copyrights to Supplemental Material that may be linked to, or accessed through, an article. The authors have granted ASM a non-exclusive, world-wide license to publish the Supplemental Material files. Please contact the corresponding author directly for reuse.

## References

[1] Azam F, Malfatti F. 2007. Microbial structuring of marine ecosystems. Nat Rev Microbiol 5:782–791. doi:10.1038/nrmicro1747 17853906

[2] Smith DC, Simon M, Alldredge AL, Azam F. 1992. Intense hydrolytic enzyme activity on marine aggregates and implications for rapid particle dissolution. Nature 359:139–142. doi:10.1038/359139a0

[3] Bidle KD, Azam F. 2001. Bacterial control of silicon regeneration from diatom detritus: significance of bacterial ectohydrolases and species identity. Limnol Oceanogr 46:1606–1623. doi:10.4319/lo.2001.46.7.1606

[4] Martinez J, Smith DC, Steward GF, Azam F. 1996. Variability in ectohydrolytic enzyme activities of pelagic marine bacteria and its significance for substrate processing in the sea. Aquat Microb Ecol 10:223–230. doi:10.3354/ame010223

[5] Grossart H-P, Czub G, Simon M. 2006. Algae–bacteria interactions and their effects on aggregation and organic matter flux in the sea. Environ Microbiol 8:1074–1084. doi:10.1111/j.1462-2920.2006.00999.x 16689728

[6] Malfatti F, Azam F. 2009. Atomic force microscopy reveals microscale networks and possible symbioses among pelagic marine bacteria. Aquat Microb Ecol 58:1–14. doi:10.3354/ame01355

[7] Biddanda B, Pomeroy L. 1988. Microbial aggregation and degradation of phytoplankton-derived detritus in seawater. Mar Ecol Prog Ser 42:79–88. doi:10.3354/meps042079

[8] Guessous G, Patsalo V, Balakrishnan R, Çağlar T, Williamson JR, Hwa T. 2023. Inherited chitinases enable sustained growth and rapid dispersal of bacteria from chitin particles. Nat Microbiol 8:1695–1705. doi:10.1038/s41564-023-01444-5 37580592 PMC13050353

[9] Fernandez VI, Yawata Y, Stocker R. 2019. A foraging mandala for aquatic microorganisms. ISME J 13:563–575. doi:10.1038/s41396-018-0309-4 30446738 PMC6461837

[10] DeLong EF, Franks DG, Alldredge AL. 1993. Phylogenetic diversity of aggregate-attached vs. free-living marine bacterial assemblages. Limnol Oceanogr 38:924–934. doi:10.4319/lo.1993.38.5.0924

[11] Dupont CL, McCrow JP, Valas R, Moustafa A, Walworth N, Goodenough U, Roth R, Hogle SL, Bai J, Johnson ZI, Mann E, Palenik B, Barbeau KA, Craig Venter J, Allen AE. 2015. Genomes and gene expression across light and productivity gradients in eastern subtropical Pacific microbial communities. ISME J 9:1076–1092. doi:10.1038/ismej.2014.198 25333462 PMC4410273

[12] García-Martínez J, Acinas SG, Massana R, Rodríguez-Valera F. 2002. Prevalence and microdiversity of Alteromonas macleodii-like microorganisms in different oceanic regions. Environ Microbiol 4:42–50. doi:10.1046/j.1462-2920.2002.00255.x 11966824

[13] Datta MS, Sliwerska E, Gore J, Polz MF, Cordero OX. 2016. Microbial interactions lead to rapid micro-scale successions on model marine particles. Nat Commun 7:11965. doi:10.1038/ncomms11965 27311813 PMC4915023

[14] McCarren J, Becker JW, Repeta DJ, Shi Y, Young CR, Malmstrom RR, Chisholm SW, DeLong EF. 2010. Microbial community transcriptomes reveal microbes and metabolic pathways associated with dissolved organic matter turnover in the sea. Proc Natl Acad Sci U S A 107:16420–16427. doi:10.1073/pnas.1010732107 20807744 PMC2944720

[15] Kiørboe T. 2001. Formation and fate of marine snow: small-scale processes with large-scale implications. Sci Mar 65:57–71. doi:10.3989/scimar.2001.65s257

[16] Grossart H-P, Tang KW, Kiørboe T, Ploug H. 2007. Comparison of cell-specific activity between free-living and attached bacteria using isolates and natural assemblages. FEMS Microbiol Lett 266:194–200. doi:10.1111/j.1574-6968.2006.00520.x 17233730

[17] Forchielli E, Sher D, Segrè D. 2022. Metabolic phenotyping of marine heterotrophs on refactored media reveals diverse metabolic adaptations and lifestyle strategies. mSystems 7:e0007022. doi:10.1128/msystems.00070-22 35856685 PMC9426600

[18] Enke TN, Leventhal GE, Metzger M, Saavedra JT, Cordero OX. 2018. Microscale ecology regulates particulate organic matter turnover in model marine microbial communities. Nat Commun 9:2743. doi:10.1038/s41467-018-05159-8 30013041 PMC6048024

[19] Pontrelli S, Szabo R, Pollak S, Schwartzman J, Ledezma-Tejeida D, Cordero OX, Sauer U. 2022. Metabolic cross-feeding structures the assembly of polysaccharide degrading communities. Sci Adv 8:eabk3076. doi:10.1126/sciadv.abk3076 35196097 PMC8865766

[20] Bassler BL, Yu C, Lee YC, Roseman S. 1991. Chitin utilization by marine bacteria. degradation and catabolism of chitin oligosaccharides by Vibrio furnissii. J Biol Chem 266:24276–24286. doi:10.1016/S0021-9258(18)54225-3 1761533

[21] Teschler JK, Zamorano-Sánchez D, Utada AS, Warner CJA, Wong GCL, Linington RG, Yildiz FH. 2015. Living in the matrix: assembly and control of Vibrio cholerae biofilms. Nat Rev Microbiol 13:255–268. doi:10.1038/nrmicro3433 25895940 PMC4437738

[22] Cai Y-M. 2020. Non-surface attached bacterial aggregates: a ubiquitous third lifestyle. Front Microbiol 11:557035. doi:10.3389/fmicb.2020.557035 33343514 PMC7746683

[23] Floyd KA, Lee CK, Xian W, Nametalla M, Valentine A, Crair B, Zhu S, Hughes HQ, Chlebek JL, Wu DC, Hwan Park J, Farhat AM, Lomba CJ, Ellison CK, Brun YV, Campos-Gomez J, Dalia AB, Liu J, Biais N, Wong GCL, Yildiz FH. 2020. c-di-GMP modulates type IV MSHA pilus retraction and surface attachment in Vibrio cholerae. Nat Commun 11:1549. doi:10.1038/s41467-020-15331-8 32214098 PMC7096442

[24] Friedman L, Kolter R. 2004. Genes involved in matrix formation in Pseudomonas aeruginosa PA14 biofilms. Mol Microbiol 51:675–690. doi:10.1046/j.1365-2958.2003.03877.x 14731271

[25] O’Neal L, Baraquet C, Suo Z, Dreifus JE, Peng Y, Raivio TL, Wozniak DJ, Harwood CS, Parsek MR. 2022. The Wsp system of Pseudomonas aeruginosa links surface sensing and cell envelope stress. Proc Natl Acad Sci U S A 119:e2117633119. doi:10.1073/pnas.2117633119 35476526 PMC9170161

[26] Yildiz F, Fong J, Sadovskaya I, Grard T, Vinogradov E. 2014. Structural characterization of the extracellular polysaccharide from Vibrio cholerae O1 El-Tor. PLoS ONE 9:e86751. doi:10.1371/journal.pone.0086751 24520310 PMC3901696

[27] Krasteva PV, Fong JCN, Shikuma NJ, Beyhan S, Navarro M, Yildiz FH, Sondermann H. 2010. Vibrio cholerae VpsT regulates matrix production and motility by directly sensing cyclic di-GMP. Science 327:866–868. doi:10.1126/science.1181185 20150502 PMC2828054

[28] Berk V, Fong JCN, Dempsey GT, Develioglu ON, Zhuang X, Liphardt J, Yildiz FH, Chu S. 2012. Molecular architecture and assembly principles of Vibrio cholerae biofilms. Science 337:236–239. doi:10.1126/science.1222981 22798614 PMC3513368

[29] Fong JC, Rogers A, Michael AK, Parsley NC, Cornell W-C, Lin Y-C, Singh PK, Hartmann R, Drescher K, Vinogradov E, Dietrich LE, Partch CL, Yildiz FH. 2017. Structural dynamics of RbmA governs plasticity of Vibrio cholerae biofilms. Elife 6:e26163. doi:10.7554/eLife.26163 28762945 PMC5605196

[30] Chiavelli DA, Marsh JW, Taylor RK. 2001. The mannose-sensitive hemagglutinin of Vibrio cholerae promotes adherence to zooplankton. Appl Environ Microbiol 67:3220–3225. doi:10.1128/AEM.67.7.3220-3225.2001 11425745 PMC93004

[31] Yu C, Lee AM, Bassler BL, Roseman S. 1991. Chitin utilization by marine bacteria. a physiological function for bacterial adhesion to immobilized carbohydrates. J Biol Chem 266:24260–24267. doi:10.1016/S0021-9258(18)54223-X 1761531

[32] Keyhani NO, Roseman S. 1996. The chitin catabolic cascade in the marine bacterium Vibrio furnissii. Molecular cloning, isolation, and characterization of a periplasmic chitodextrinase. J Biol Chem 271:33414–33424. doi:10.1074/jbc.271.52.33414 8969204

[33] Yildiz FH, Visick KL. 2009. Vibrio biofilms: so much the same yet so different. Trends Microbiol. 17:109–118. doi:10.1016/j.tim.2008.12.004 19231189 PMC2729562

[34] Schwartzman JA, Ebrahimi A, Chadwick G, Sato Y, Roller BRK, Orphan VJ, Cordero OX. 2022. Bacterial growth in multicellular aggregates leads to the emergence of complex life cycles. Curr Biol 32:3059–3069. doi:10.1016/j.cub.2022.06.011 35777363 PMC9496226

[35] Alhede M, Kragh KN, Qvortrup K, Allesen-Holm M, van Gennip M, Christensen LD, Jensen PØ, Nielsen AK, Parsek M, Wozniak D, Molin S, Tolker-Nielsen T, Høiby N, Givskov M, Bjarnsholt T. 2011. Phenotypes of non-attached Pseudomonas aeruginosa aggregates resemble surface attached biofilm. PLoS ONE 6:e27943. doi:10.1371/journal.pone.0027943 22132176 PMC3221681

[36] Melaugh G, Martinez VA, Baker P, Hill PJ, Howell PL, Wozniak DJ, Allen RJ. 2023. Distinct types of multicellular aggregates in Pseudomonas aeruginosa liquid cultures. NPJ Biofilms Microbiomes 9:1–14. doi:10.1038/s41522-023-00412-5 37507436 PMC10382557

[37] Wang D, Xu X, Deng X, Chen C, Li B, Tan H, Wang H, Tang S, Qiu H, Chen J, Ke B, Ke C, Kan B. 2010. Detection of Vibrio cholerae O1 and O139 in environmental water samples by an immunofluorescent-aggregation assay. Appl Environ Microbiol 76:5520–5525. doi:10.1128/AEM.02559-09 20581193 PMC2918967

[38] Silva AJ, Benitez JA. 2016. Vibrio cholerae biofilms and cholera pathogenesis. PLOS Negl Trop Dis 10:e0004330. doi:10.1371/journal.pntd.0004330 26845681 PMC4741415

[39] Jackson KD, Starkey M, Kremer S, Parsek MR, Wozniak DJ. 2004. Identification of psl, a locus encoding a potential exopolysaccharide that is essential for Pseudomonas aeruginosa PAO1 biofilm formation. J Bacteriol 186:4466–4475. doi:10.1128/JB.186.14.4466-4475.2004 15231778 PMC438565

[40] Ma L, Jackson KD, Landry RM, Parsek MR, Wozniak DJ. 2006. Analysis of Pseudomonas aeruginosa conditional Psl variants reveals roles for the Psl polysaccharide in adhesion and maintaining biofilm structure postattachment . J Bacteriol 188:8213–8221. doi:10.1128/JB.01202-06 16980452 PMC1698210

[41] Jemielita M, Wingreen NS, Bassler BL. 2018. Quorum sensing controls Vibrio cholerae multicellular aggregate formation. Elife 7:e42057. doi:10.7554/eLife.42057 30582742 PMC6351105

[42] Chiang SL, Taylor RK, Koomey M, Mekalanos JJ. 1995. Single amino acid substitutions in the N-terminus of Vibrio cholerae TcpA affect colonization, autoagglutination, and serum resistance. Mol Microbiol 17:1133–1142. doi:10.1111/j.1365-2958.1995.mmi_17061133.x 8594332

[43] Nayfach S, Rodriguez-Mueller B, Garud N, Pollard KS. 2016. An integrated metagenomics pipeline for strain profiling reveals novel patterns of bacterial transmission and biogeography. Genome Res 26:1612–1625. doi:10.1101/gr.201863.115 27803195 PMC5088602

[44] Ivars-Martinez E, Martin-Cuadrado A-B, D’Auria G, Mira A, Ferriera S, Johnson J, Friedman R, Rodriguez-Valera F. 2008. Comparative genomics of two ecotypes of the marine planktonic copiotroph Alteromonas macleodii suggests alternative lifestyles associated with different kinds of particulate organic matter. ISME J 2:1194–1212. doi:10.1038/ismej.2008.74 18670397

[45] Mitulla M, Dinasquet J, Guillemette R, Simon M, Azam F, Wietz M. 2016. Response of bacterial communities from California coastal waters to alginate particles and an alginolytic Alteromonas macleodii strain. Environ Microbiol 18:4369–4377. doi:10.1111/1462-2920.13314 27059936

[46] Wietz M, López-Pérez M, Sher D, Biller SJ, Rodriguez-Valera F. 2022. Microbe profile: Alteromonas macleodii − a widespread, fast-responding, ‘interactive’ marine bacterium. Microbiology (Reading) 168:001236. doi:10.1099/mic.0.001236 36748580

[47] Koch H, Germscheid N, Freese HM, Noriega-Ortega B, Lücking D, Berger M, Qiu G, Marzinelli EM, Campbell AH, Steinberg PD, Overmann J, Dittmar T, Simon M, Wietz M. 2020. Genomic, metabolic and phenotypic variability shapes ecological differentiation and Intraspecies interactions of Alteromonas macleodii. Sci Rep 10:1–14. doi:10.1038/s41598-020-57526-5 31964928 PMC6972757

[48] Hennon GM, Morris JJ, Haley ST, Zinser ER, Durrant AR, Entwistle E, Dokland T, Dyhrman ST. 2018. The impact of elevated CO_2_ on Prochlorococcus and microbial interactions with ‘helper’ bacterium Alteromonas. ISME J 12:520–531. doi:10.1038/ismej.2017.189 29087378 PMC5776468

[49] Manck LE, Park J, Tully BJ, Poire AM, Bundy RM, Dupont CL, Barbeau KA. 2022. Petrobactin, a siderophore produced by Alteromonas, mediates community iron acquisition in the global ocean. ISME J 16:358–369. doi:10.1038/s41396-021-01065-y 34341506 PMC8776838

[50] Manck LE, Espinoza JL, Dupont CL, Barbeau KA. 2020. Transcriptomic study of substrate-specific transport mechanisms for iron and carbon in the marine copiotroph Alteromonas macleodii. mSystems 5:e00070-20. doi:10.1128/mSystems.00070-20 32345736 PMC7190382

[51] Baumann L, Baumann P, Mandel M, Allen RD. 1972. Taxonomy of aerobic marine eubacteria. J Bacteriol 110:402–429. doi:10.1128/jb.110.1.402-429.1972 4552999 PMC247423

[52] Sher D, George EE, Wietz M, Gifford S, Zoccaratto L, Weissberg O, Koedooder C, Waseem BV, Filho MMB, Mireles R, Malavin S, Naim ML, Idan T, Shrivastava V, Itelson L, Sade D, Hamoud AA, Soussan Y, Barak N, Karp P, Moore L. 2023. Collaborative metabolic curation of an emerging model marine bacterium, Alteromonas macleodii ATCC 27126. bioRxiv. doi:10.1101/2023.12.13.571488 PMC12021255 40273159

[53] Keseler IM, Gama-Castro S, Mackie A, Billington R, Bonavides-Martínez C, Caspi R, Kothari A, Krummenacker M, Midford PE, Muñiz-Rascado L, Ong WK, Paley S, Santos-Zavaleta A, Subhraveti P, Tierrafría VH, Wolfe AJ, Collado-Vides J, Paulsen IT, Karp PD. 2021. The EcoCyc database in 2021. Front Microbiol 12:711077. doi:10.3389/fmicb.2021.711077 34394059 PMC8357350

[54] Karp PD, Billington R, Caspi R, Fulcher CA, Latendresse M, Kothari A, Keseler IM, Krummenacker M, Midford PE, Ong Q, Ong WK, Paley SM, Subhraveti P. 2019. The BioCyc collection of microbial genomes and metabolic pathways. Brief Bioinform 20:1085–1093. doi:10.1093/bib/bbx085 29447345 PMC6781571

[55] Nesper J, Lauriano CM, Klose KE, Kapfhammer D, Kraiss A, Reidl J. 2001. Characterization of Vibrio cholerae O1 El Tor galU and galE mutants: influence on lipopolysaccharide structure, colonization, and biofilm formation. Infect Immun 69:435–445. doi:10.1128/IAI.69.1.435-445.2001 11119535 PMC97901

[56] Møller T, Franch T, Udesen C, Gerdes K, Valentin-Hansen P. 2002. Spot 42 RNA mediates discoordinate expression of the E. coli galactose operon. Genes Dev 16:1696–1706. doi:10.1101/gad.231702 12101127 PMC186370

[57] Bækkedal C, Haugen P. 2015. The spot 42 RNA: a regulatory small RNA with roles in the central metabolism. RNA Biol 12:1071–1077. doi:10.1080/15476286.2015.1086867 26327359 PMC4829326

[58] Agarwal S, Gopal K, Chhabra G, Dixit A. 2009. Molecular cloning, sequence analysis and homology modeling of galE encoding UDP-galactose 4-epimerase of Aeromonas hydrophila. Bioinformation 4:216–222. doi:10.6026/97320630004216 20461162 PMC2859578

[59] Li C-T, Liao C-T, Du S-C, Hsiao Y-P, Lo H-H, Hsiao Y-M. 2014. Functional characterization and transcriptional analysis of galE gene encoding a UDP-galactose 4-epimerase in Xanthomonas campestris pv. campestris. Microbiol Res 169:441–452. doi:10.1016/j.micres.2013.08.005 24120348

[60] Frey PA, Hegeman AD. 2013. Chemical and stereochemical actions of UDP–galactose 4-epimerase. Acc Chem Res 46:1417–1426. doi:10.1021/ar300246k 23339688

[61] Gossen JA, Molijn AC, Douglas GR, Vijg J. 1992. Application of galactose-sensitive E.coli strains as selective hosts for LacZ^−^ plasmids. Nucleic Acids Res 20:3254. doi:10.1093/nar/20.12.3254 1620626 PMC312470

[62] Chai Y, Beauregard PB, Vlamakis H, Losick R, Kolter R. 2012. Galactose metabolism plays a crucial role in biofilm formation by Bacillus subtilis. mBio 3:e00184-12. doi:10.1128/mBio.00184-12 22893383 PMC3419520

[63] Diner RE, Schwenck SM, McCrow JP, Zheng H, Allen AE. 2016. Genetic manipulation of competition for nitrate between heterotrophic bacteria and diatoms. Front Microbiol 7:880. doi:10.3389/fmicb.2016.00880 27375600 PMC4899447

[64] Bowman JP, McMeekin TA. 2015. *Alteromonas*, p 1–7. In Bergey’s manual of systematics of archaea and bacteria. American Cancer Society.

[65] Daniels M, van Vliet S, Ackermann M. 2023. Changes in interactions over ecological time scales influence single-cell growth dynamics in a metabolically coupled marine microbial community. ISME J 17:406–416. doi:10.1038/s41396-022-01312-w 36611102 PMC9938273

[66] Passow U, Alldredge AL. 1995. A dye-binding assay for the spectrophotometric measurement of transparent exopolymer particles (TEP). Limnol Oceanogr 40:1326–1335. doi:10.4319/lo.1995.40.7.1326

[67] Bittar TB, Passow U, Hamaraty L, Bidle KD, Harvey EL. 2018. An updated method for the calibration of transparent exopolymer particle measurements. Limnol Ocean Methods 16:621–628. doi:10.1002/lom3.10268

[68] Villar E, Vannier T, Vernette C, Lescot M, Cuenca M, Alexandre A, Bachelerie P, Rosnet T, Pelletier E, Sunagawa S, Hingamp P. 2018. The Ocean Gene Atlas: exploring the biogeography of plankton genes online. Nucleic Acids Res. 46:W289–W295. doi:10.1093/nar/gky376 29788376 PMC6030836

[69] Vernette C, Lecubin J, Sánchez P, Coordinators TO, Sunagawa S, Delmont TO, Acinas SG, Pelletier E, Hingamp P, Lescot M. 2022. The Ocean Gene Atlas v2.0: online exploration of the biogeography and phylogeny of plankton genes. Nucleic Acids Res. 50:W516–W526. doi:10.1093/nar/gkac420 35687095 PMC9252727

[70] Nakao R, Senpuku H, Watanabe H. 2006. Porphyromonas gingivalis galE is involved in lipopolysaccharide O-antigen synthesis and biofilm formation. Infect Immun 74:6145–6153. doi:10.1128/IAI.00261-06 16954395 PMC1695533

[71] Niou Y-K, Wu W-L, Lin L-C, Yu M-S, Shu H-Y, Yang H-H, Lin G-H. 2009. Role of galE on biofilm formation by Thermus spp. Biochem Biophys Res Commun 390:313–318. doi:10.1016/j.bbrc.2009.09.120 19800315

[72] Raguénès GH, Peres A, Ruimy R, Pignet P, Christen R, Loaec M, Rougeaux H, Barbier G, Guezennec JG. 1997. Alteromonas infernus sp. nov., a new polysaccharide-producing bacterium isolated from a deep-sea hydrothermal vent. J Appl Microbiol 82:422–430. doi:10.1046/j.1365-2672.1997.00125.x 9134716

[73] Concórdio-Reis P, Alves VD, Moppert X, Guézennec J, Freitas F, Reis MAM. 2021. Characterization and biotechnological potential of extracellular polysaccharides synthesized by Alteromonas strains isolated from French polynesia marine environments. Mar Drugs 19:522. doi:10.3390/md19090522 34564184 PMC8470090

[74] Liparoti V, Molinaro A, Sturiale L, Garozzo D, Nazarenko EL, Gorshkova RP, Ivanova EP, Shevcenko LS, Lanzetta R, Parrilli M. 2006. Structural analysis of the deep rough lipopolysaccharide from Gram negative bacterium Alteromonas macleodii ATCC 27126T: the first finding of β-Kdo in the inner core of lipopolysaccharides . Eur J Org Chem 2006:4710–4716. doi:10.1002/ejoc.200600489

[75] Gonzaga A, Martin-Cuadrado A-B, López-Pérez M, Megumi Mizuno C, García-Heredia I, Kimes NE, Lopez-García P, Moreira D, Ussery D, Zaballos M, Ghai R, Rodriguez-Valera F. 2012. Polyclonality of concurrent natural populations of Alteromonas macleodii. Genome Biol Evol 4:1360–1374. doi:10.1093/gbe/evs112 23212172 PMC3542563

[76] López-Pérez M, Rodriguez-Valera F. 2016. Pangenome evolution in the marine bacterium Alteromonas. Genome Biol Evol 8:1556–1570. doi:10.1093/gbe/evw098 27189983 PMC4898812

[77] Ivanova EP, López-Pérez M, Zabalos M, Nguyen SH, Webb HK, Ryan J, Lagutin K, Vyssotski M, Crawford RJ, Rodriguez-Valera F. 2015. Ecophysiological diversity of a novel member of the genus Alteromonas, and description of Alteromonas mediterranea sp. nov. Antonie Van Leeuwenhoek 107:119–132. doi:10.1007/s10482-014-0309-y 25326795

[78] Burd AB, Jackson GA. 2009. Particle aggregation. Annu Rev Mar Sci 1:65–90. doi:10.1146/annurev.marine.010908.163904 21141030

[79] Sorroche FG, Spesia MB, Zorreguieta A, Giordano W. 2012. A positive correlation between bacterial autoaggregation and biofilm formation in native Sinorhizobium meliloti isolates from Argentina. Appl Environ Microbiol 78:4092–4101. doi:10.1128/AEM.07826-11 22492433 PMC3370541

[80] DePas WH, Bergkessel M, Newman DK. 2019. Aggregation of nnontuberculous mycobacteria is regulated by carbon-nitrogen balance. mBio 10:e01715-19. doi:10.1128/mBio.01715-19 31409683 PMC6692514

[81] Lee E-M, Ahn S-H, Park J-H, Lee J-H, Ahn S-C, Kong I-S. 2004. Identification of oligopeptide permease (opp) gene cluster in Vibrio fluvialis and characterization of biofilm production by oppA knockout mutation. FEMS Microbiol Lett 240:21–30. doi:10.1016/j.femsle.2004.09.007 15500975

[82] Eilers H, Pernthaler J, Amann R. 2000. Succession of pelagic marine bacteria during enrichment: a close look at cultivation-induced shifts. Appl Environ Microbiol 66:4634–4640. doi:10.1128/AEM.66.11.4634-4640.2000 11055904 PMC92360

[83] Nystrom T, Albertson N, Kjelleberg S. 1988. Synthesis of membrane and periplasmic proteins during starvation of a marine Vibrio sp. Microbiology 134:1645–1651. doi:10.1099/00221287-134-6-1645 3221201

[84] Amy PS, Pauling C, Morita RY. 1983. Starvation-survival processes of a marine Vibrio. Appl Environ Microbiol 45:1041–1048. doi:10.1128/aem.45.3.1041-1048.1983 16346228 PMC242407

[85] Ducklow H, Steinberg D, Buesseler K. 2001. Upper ocean carbon export and the biological pump. Oceanography 14:50–58. doi:10.5670/oceanog.2001.06

[86] De La Rocha CL, Passow U. 2007. Factors influencing the sinking of POC and the efficiency of the biological carbon pump. Deep-Sea Res II: Top Stud Oceanogr 54:639–658. doi:10.1016/j.dsr2.2007.01.004

[87] Alldredge AL, Passow U, Logan BE. 1993. The abundance and significance of a class of large, transparent organic particles in the ocean. Deep-Sea Res I: Oceanogr Res Papers 40:1131–1140. doi:10.1016/0967-0637(93)90129-Q

[88] Passow U. 2002. Transparent exopolymer particles (TEP) in aquatic environments. Prog Oceanogr 55:287–333. doi:10.1016/S0079-6611(02)00138-6

[89] Stoderegger KE, Herndl GJ. 1999. Production of exopolymer particles by marine bacterioplankton under contrasting turbulence conditions. Mar Ecol Prog Ser 189:9–16. doi:10.3354/meps189009

[90] Passow U. 2002. Production of transparent exopolymer particles (TEP) by phyto- and bacterioplankton. Mar Ecol Prog Ser 236:1–12. doi:10.3354/meps236001

[91] Radić T, Ivancić I, Fuks D, Radić J. 2006. Marine bacterioplankton production of polysaccharidic and proteinaceous particles under different nutrient regimes. FEMS Microbiol Ecol 58:333–342. doi:10.1111/j.1574-6941.2006.00176.x 17117978

[92] Passow U, Alldredge AL. 1994. Distribution, size and bacterial colonization of transparent exopolymer particles (TEP) in the ocean. Mar Ecol Prog Ser 113:185–198. doi:10.3354/meps113185

[93] Gonzaga A, López-Pérez M, Martin-Cuadrado A-B, Ghai R, Rodriguez-Valera F. 2012. Complete genome sequence of the copiotrophic marine bacterium Alteromonas macleodii strain ATCC 27126T. J Bacteriol 194:6998–6998. doi:10.1128/JB.01565-12 23209244 PMC3510622

[94] Hou S, López-Pérez M, Pfreundt U, Belkin N, Stüber K, Huettel B, Reinhardt R, Berman-Frank I, Rodriguez-Valera F, Hess WR. 2018. Benefit from decline: the primary transcriptome of Alteromonas macleodii str. Te101 during Trichodesmium demise. ISME J 12:981–996. doi:10.1038/s41396-017-0034-4 29335641 PMC5864184

[95] Koch H, Dürwald A, Schweder T, Noriega-Ortega B, Vidal-Melgosa S, Hehemann J-H, Dittmar T, Freese HM, Becher D, Simon M, Wietz M. 2019. Biphasic cellular adaptations and ecological implications of Alteromonas macleodii degrading a mixture of algal polysaccharides. ISME J 13:92–103. doi:10.1038/s41396-018-0252-4 30116038 PMC6298977

[96] Pedler BE, Aluwihare LI, Azam F. 2014. Single bacterial strain capable of significant contribution to carbon cycling in the surface ocean. Proc Natl Acad Sci U S A 111:7202–7207. doi:10.1073/pnas.1401887111 24733921 PMC4034236

[97] Pedler Sherwood B, Shaffer EA, Reyes K, Longnecker K, Aluwihare LI, Azam F. 2015. Metabolic characterization of a model heterotrophic bacterium capable of significant chemical alteration of marine dissolved organic matter. Mar Chem 177:357–365. doi:10.1016/j.marchem.2015.06.027

[98] López-Pérez M, Gonzaga A, Ivanova EP, Rodriguez-Valera F. 2014. Genomes of Alteromonas australica, a world apart. BMC Genomics 15:483. doi:10.1186/1471-2164-15-483 24942065 PMC4119200

[99] Amarnath K, Narla AV, Pontrelli S, Dong J, Reddan J, Taylor BR, Caglar T, Schwartzman J, Sauer U, Cordero OX, Hwa T. 2023. Stress-induced metabolic exchanges between complementary bacterial types underly a dynamic mechanism of inter-species stress resistance. Nat Commun 14:3165. doi:10.1038/s41467-023-38913-8 37258505 PMC10232422

[100] Gurevich A, Saveliev V, Vyahhi N, Tesler G. 2013. QUAST: quality assessment tool for genome assemblies. Bioinformatics 29:1072–1075. doi:10.1093/bioinformatics/btt086 23422339 PMC3624806

[101] Aziz RK, Bartels D, Best AA, DeJongh M, Disz T, Edwards RA, Formsma K, Gerdes S, Glass EM, Kubal M, et al.. 2008. The RAST server: rapid annotations using subsystems technology. BMC Genomics 9:75. doi:10.1186/1471-2164-9-75 18261238 PMC2265698

[102] Overbeek R, Olson R, Pusch GD, Olsen GJ, Davis JJ, Disz T, Edwards RA, Gerdes S, Parrello B, Shukla M, Vonstein V, Wattam AR, Xia F, Stevens R. 2014. The SEED and the rapid annotation of microbial genomes using subsystems technology (RAST). Nucleic Acids Res. 42:D206–D214. doi:10.1093/nar/gkt1226 24293654 PMC3965101

[103] Brettin T, Davis JJ, Disz T, Edwards RA, Gerdes S, Olsen GJ, Olson R, Overbeek R, Parrello B, Pusch GD, Shukla M, Thomason JA, Stevens R, Vonstein V, Wattam AR, Xia F. 2015. RASTtk: a modular and extensible implementation of the RAST algorithm for building custom annotation pipelines and annotating batches of genomes. Sci Rep 5:8365. doi:10.1038/srep08365 25666585 PMC4322359

[104] Pollak B, Matute T, Nuñez I, Cerda A, Lopez C, Vargas V, Kan A, Bielinski V, von Dassow P, Dupont CL, Federici F. 2020. Universal loop assembly: open, efficient and cross-kingdom DNA fabrication. Synth Biol (Oxf) 5:ysaa001. doi:10.1093/synbio/ysaa001 32161816 PMC7052795

[105] Strand TA, Lale R, Degnes KF, Lando M, Valla S. 2014. A new and improved host-independent plasmid system for RK2-based conjugal transfer. PLoS One 9:e90372. doi:10.1371/journal.pone.0090372 24595202 PMC3940858

[106] Wick R. 2024. rrwick/Filtlong. C++

[107] Li H. 2016. Minimap and miniasm: fast mapping and de novo assembly for noisy long sequences. Bioinformatics 32:2103–2110. doi:10.1093/bioinformatics/btw152 27153593 PMC4937194

[108] Kolmogorov M, Yuan J, Lin Y, Pevzner PA. 2019. Assembly of long, error-prone reads using repeat graphs. Nat Biotechnol 37:540–546. doi:10.1038/s41587-019-0072-8 30936562

[109] Schwengers O, Jelonek L, Dieckmann MA, Beyvers S, Blom J, Goesmann A. 2021. Bakta: rapid and standardized annotation of bacterial genomes via alignment-free sequence identification. Microb Genom 7:000685. doi:10.1099/mgen.0.000685 34739369 PMC8743544

[110] Parks DH, Imelfort M, Skennerton CT, Hugenholtz P, Tyson GW. 2015. CheckM: assessing the quality of microbial genomes recovered from isolates, single cells, and metagenomes. Genome Res 25:1043–1055. doi:10.1101/gr.186072.114 25977477 PMC4484387

[111] Wick RR, Schultz MB, Zobel J, Holt KE. 2015. Bandage: interactive visualization of de novo genome assemblies. Bioinformatics 31:3350–3352. doi:10.1093/bioinformatics/btv383 26099265 PMC4595904

[112] van der Walt S, Schönberger JL, Nunez-Iglesias J, Boulogne F, Warner JD, Yager N, Gouillart E, Yu T. 2014. scikit-image: image processing in Python. PeerJ 2:e453. doi:10.7717/peerj.453 25024921 PMC4081273

