# Supplementary material for: Marine bacteria Alteromonas spp. require UDP-glucose-4-epimerase for aggregation and production of sticky exopolymer
Source: mBio. 2024 Jul 3;15(8):e00038-24. doi: 10.1128/mbio.00038-24 (PMC11325263; doi:10.1128/mbio.00038-24)
Supplement: Supplemental material — Fig. S1 to S5 and Table S1. [file mbio.00038-24-s0001.pdf]

Supplemental material for Robertson *et al*. (2024) “Marine Bacteria *Alteromonas* spp. Require UDP-glucose-4-epimerase for Aggregation and Production of Sticky Exopolymer”

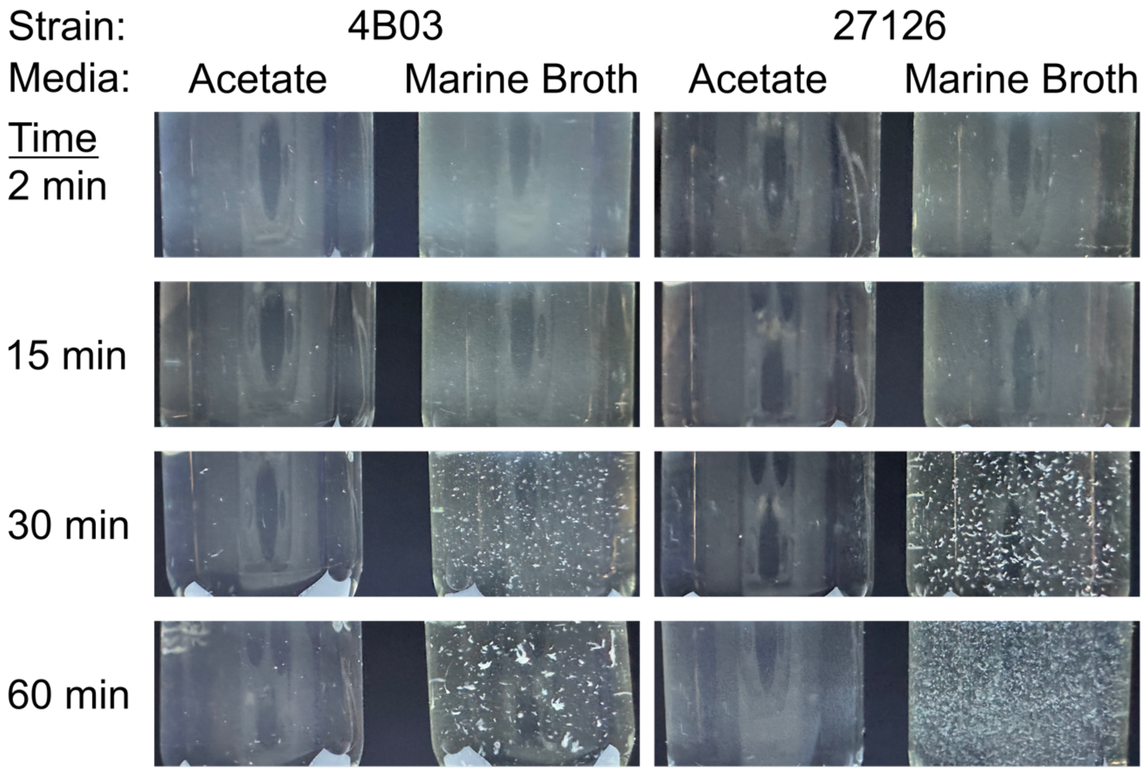

Figure S1. Aggregation of 4B03 and 27126 in Marine Broth following transfer from planktonic preculture. Acetate precultures in late exponential growth (OD ≈0.7) were resuspended and diluted 1:10 in pre-warmed acetate or Marine Broth, then shaken, slanted, and briefly removed to photograph during the first hour; see Materials and Methods for details. Images are taken from the side of 18mm test tubes, lit from beneath by an LED light panel. Images are cropped to remove glare on the bottom of the tube and at the liquid-air interface. Some glare is still evident as whitish triangles on the bottom of the tube, these are from the corners of the light panel.

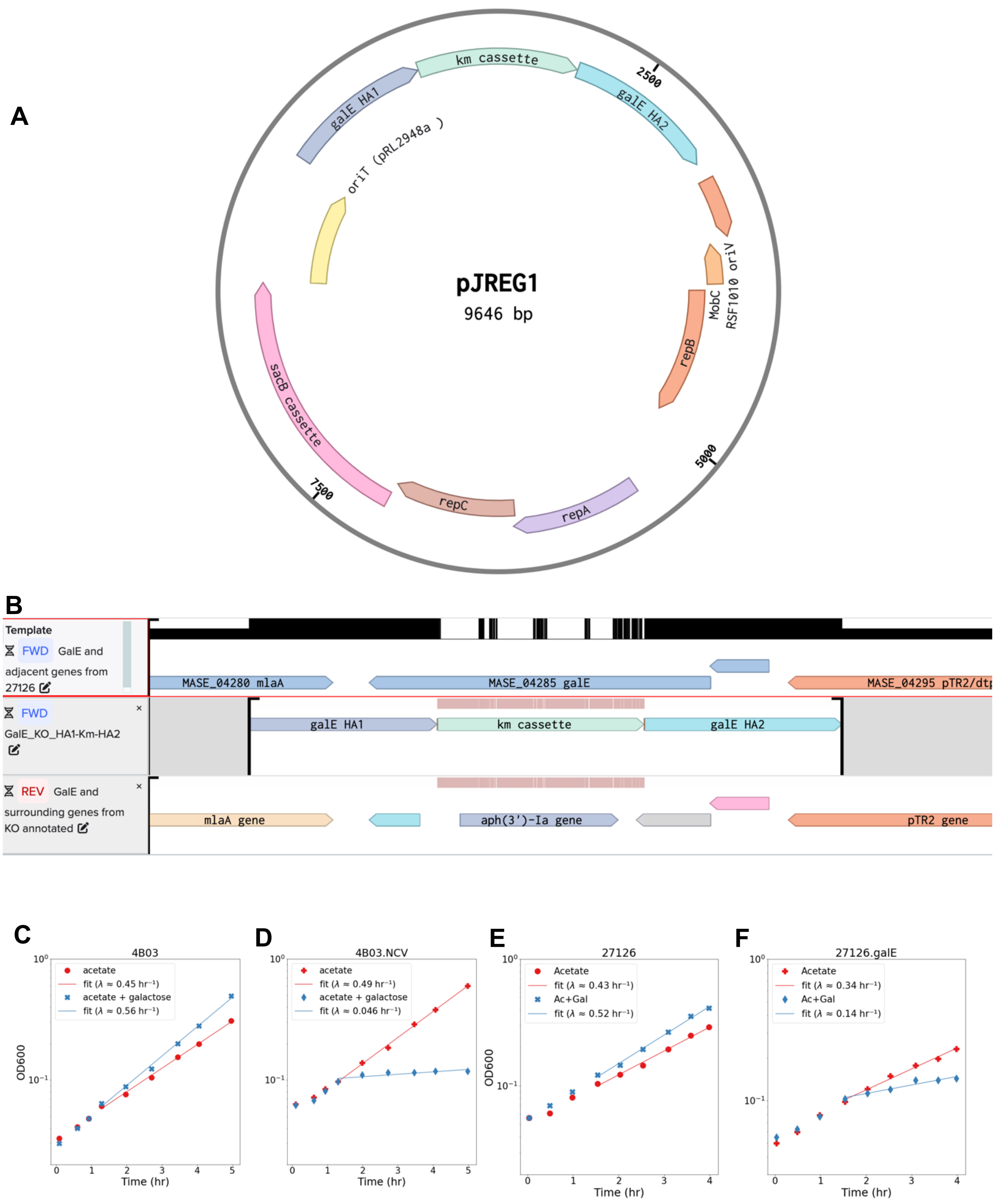

Figure S2. Construction and validation of Δ*galE*::*kan*^{r} mutation in strain 27126.*galE*. (A) Plasmid map of pJREG1, created to disrupt *galE* in 27126 with a kanamycin resistance cassette guided by homology arms HA1 and HA2. (B) Multiple sequence

alignment of *galE* and surrounding genes in 27126 (top row) vs 27126.*galE* (bottom row). Middle row shows the portion of pJREG1 containing the Kanamycin cassette and homology arms HA1 and HA2. Nucleotide identity is shown in black along the top of the first row, and regions of sequence divergence with respect to the template are shown in pale red along the top of subsequent rows. In the top row, locus tags are based on the 27126 genome and gene names are based off protein similarity to *E. coli* and *S. cerevisiae*. In the bottom row, gene names were automatically annotated by Plasmidsaurus using Bakta (1). (C-F) Growth curves of (C) 4B03 and (D) 4B03.NCV in MBL + acetate (40mM) with or without added galactose (10mM). Cultures (5ml) were started from precultures growing exponentially in MBL +acetate for at least 10 doublings to an OD between 0.5 and 1.0.

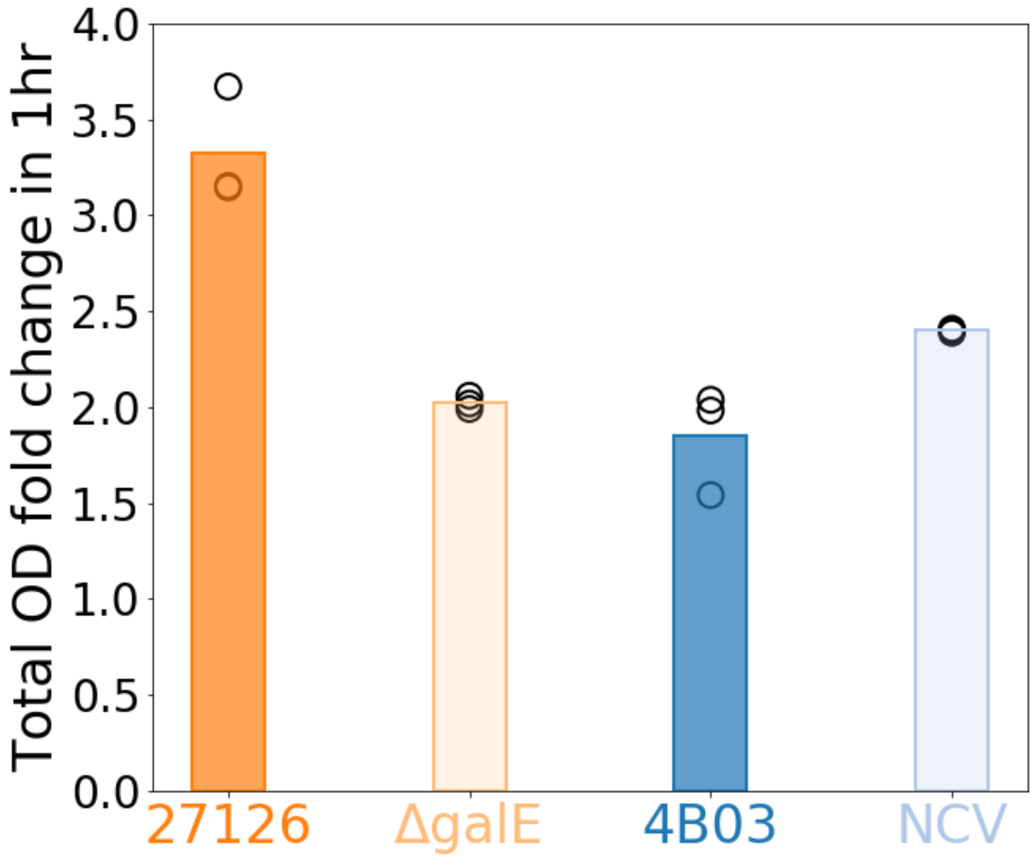

Figure S3. Growth of each strain in Marine Broth 1 hour after transfer from acetate pre-culture. Growth is shown as relative OD, or (OD at 1h)/(OD at inoculation), such that a value of 1.0 would indicate no growth.

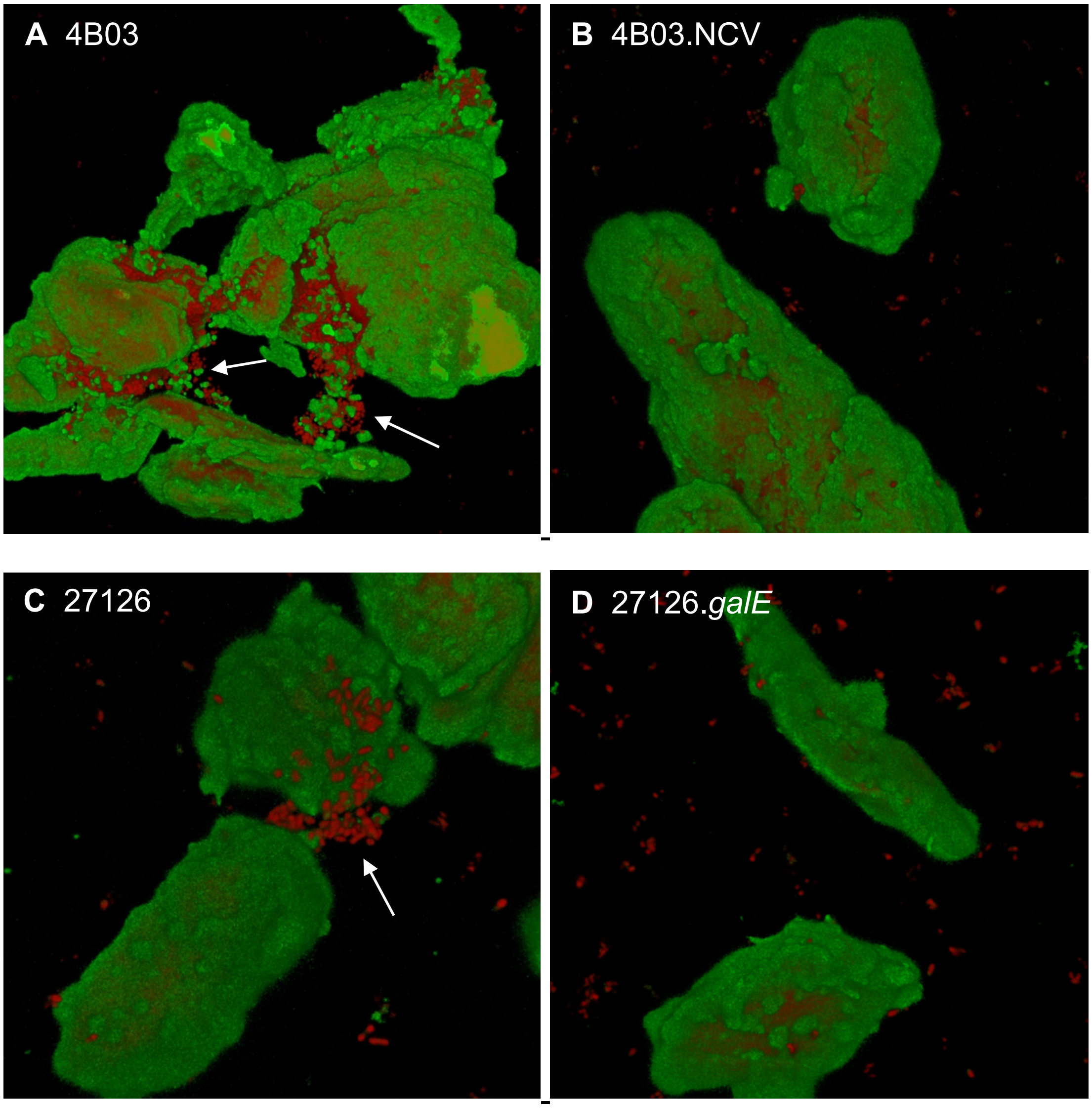

Figure S4. 3D projections of Z-stack confocal microscopic images of chitin particles (green, WGA-Fluorescein) and cells (red, Syto60) showing examples of cells in aggregates with chitin particles (A- 4B03; C- 27126) or chitin particles without cells aggregating (B-4B03.NCV; D-27126.galE). Scales differ but can be compared by FOV width: A-125µm, B-90µm, C-50µm, D-75µm. Note that chitin particles also take up Syto60, so some red fluorescence bleeds through from under the green WGA-FITC signal. Attached cells can be differentiated from bleed through by their appearance: they individually look like red grains of rice and in aggregates resemble loose balls of rice (marked by white arrows in A and C).

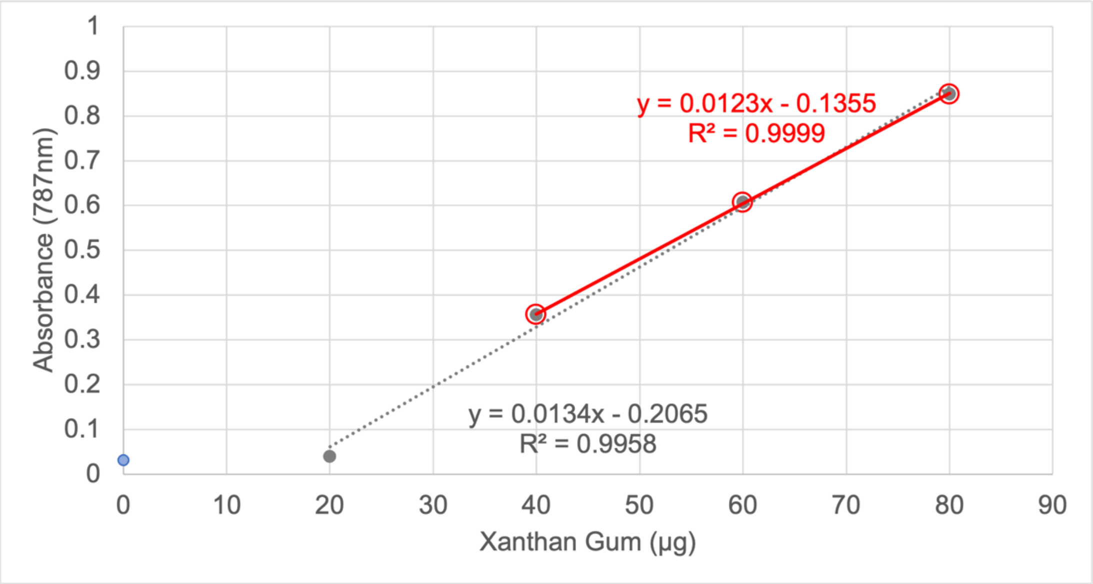

Figure S5. Xanthan gum standard curve for TEP measurements. Xanthan gum (Sigma G1253) was dissolved in milliQ water at 80 mg/l, then dilutions were prepared at 1ml final volume in capped polypropylene tubes. Then, 0.5ml of AB staining mix was mixed added to each tube and mixed by vortexing, leading to the precipitation of xanthan gum with the stain. Samples were poured onto 0.4 µm pore polycarbonate filters and separated by low vacuum, then retained stain was eluted with 80% sulfuric acid and absorbance was measured at 787nm. Fit lines excluded 0 ug point because of high background: the gray equation and dotted line show the fit for points 20-80µg, and the solid red line and equation show the fit for points 40-80µg. The fit of the solid red line was used to convert absorbance values to µg xanthan gum equivalents in Figure 6. A negative y-intercept is common in this standard curve method (Bittar *et al*., 2018) (2).

Table S1. Survey of *galE* gene copy number and operon characteristics across select members of class *Gammaproteobacteria*.

Order	Family	Genus	Species/strain	galE copies	in an operon?	Alteromonas type? (Fig S2B)	E.coli type? (Fig 2B)	Other gene(s) in operon	Note
alteromonadales	Alteromonadaceae	Alteromonas	macelodii ATCC 27126	1	yes	yes	no	DUF6170 family protein	
alteromonadales	Alteromonadaceae	Alteromonas	sp. ALT199/4B03	1	yes	yes	no	hypothetical protein	
alteromonadales	Alteromonadaceae	Alteromonas	australica H 17	1	yes	yes	no	hypothetical protein	
alteromonadales	Alteromonadaceae	Alteromonas	stellipolaris	1	yes	yes	no	hypothetical protein	
alteromonadales	Alteromonadaceae	Alteromonas	mediterranea DE	2*	yes	yes	no	hypothetical protein	*2nd copy in operon w/ DUF4982 and hypothetical protein
alteromonadales	Alteromonadaceae	Salinimonas	chungwhensis DSM 16280	1	yes	yes	no	hypothetical protein	
alteromonadales	Alteromonadaceae	Salinimonas	lutimaris DPSR-4	1	yes	yes	no	DUF6170 family protein	
alteromonadales	Alteromonadaceae	Paraglaciecola	polaris LMG 21857	1	yes	yes	no	hypothetical protein	
alteromonadales	Alteromonadaceae	Paraglaciecola	mesophila GPM4	1	yes	yes	no	hypothetical protein	
alteromonadales	Alteromonadaceae	Paraglaciecola	psychrophila 170	1	yes	yes*	no	hypothetical protein	*small insertion of 2-gene operon before vacJ/mlaA
alteromonadales	Alteromonadaceae	Glaciecola	pallidula DSM 14239	1	no*	yes	no	-	*DUF6170 family protein adjacent, but annotated as separate operon
alteromonadales	Alteromonadaceae	Aestuariibacter	aggregatus	2*	yes	yes	no	hypothetical protein	*2nd copy in another operon w/ glycoside hydrolase and hyp prot
alteromonadales	Pseudoalteromonadaceae	Pseudoalteromonas	tunicata D2	1	no*	-	-	-	*putative capsule operon upstream
alteromonadales	Pseudoalteromonadaceae	Pseudoalteromonas	aurantia 208	1	no	-	-	-	
alteromonadales	Pseudoalteromonadaceae	Pseudoalteromonas	haloplanktis TAC125	1	no	-	-	-	
alteromonadales	Pseudoalteromonadaceae	Pseudoalteromonas	sp. SM9913	1	no	-	-	-	
alteromonadales	Shewanellaceae	Shewanella	oneidensis MR-1	1	yes	no	no	galU	
alteromonadales	Shewanellaceae	Shewanella	algicola JCM 31092	1	no	-	-	-	
alteromonadales	Shewanellaceae	Shewanella	colwelliana ATCC 39565	1	yes	no		galU	
alteromonadales	Psychromonadaceae	Psychromonas	arctica DSM 14288	2	yes	no	yes	galT, galK, galM	
alteromonadales	Psychromonadaceae	Psychromonas	sp. 6C06	2	yes	no	no	UDP-GalNActransferase	
alteromonadales	Idiomarinaceae	Idiomarina	woesei DSM 27808	1	no	-	-	-	
alteromonadales	Idiomarinaceae	Idiomarina	atlantica MCCC 1A10513	1	yes	no	no	S8 family serine protease	
vibrionales	Vibrionaceae	Vibrio	cholerae O1 El Tor N16961	1	no	-	-	-	
vibrionales	Vibrionaceae	Vibrio	parahaemolyticus	2*	yes	no	yes	galT, galK, galM	*2nd copy not in operon
vibrionales	Vibrionaceae	Vibrio	alginolyticus ATCC 17749	1	no	-	-	-	
vibrionales	Vibrionaceae	Vibrio	splendidus 1A01	1	yes	no	yes	galT, galK, galM	
pseudomonadales	Marinobacteraceae	Marinobacter	adhaerens HP15	1	yes	no	no	guaA, guaB, tadA, UDP-glucose dehydrogenase, HIT protein	
pseudomonadales	Marinobacteraceae	Marinobacter	lipolyticus SM19	1	yes	no	no	tadA	
pseudomonadales	Marinobacteraceae	Marinobacter	algicola DG893	1	yes	no	no	guaA, guaB, tadA	
pseudomonadales	Pseudomonadaceae	Pseudomonas	putida KT2440	1	no*	-	-	-	*between two operons encoding genes for EPS/alginate production
pseudomonadales	Pseudomonadaceae	Pseudomonas	aeruginosa ATCC 15692	1	yes	no	no	glycosyl transferase, ABC transporter ATP-binding protein	
aeromonadales	Aeromonadaceae	Aeromonas	hydrophila ATCC 7966	2*	yes	no	yes	galT, galK, galM	*2nd copy not in operon
aeromonadales	Aeromonadaceae	Aeromonas	salmonicida O23A	2*	yes	no	yes	galT, galK, galM	*2nd copy not in operon
enterobacterales	Enterobacteriaceae	Escherichia	coli K-12 substr. MG1655	1	yes	no	yes	galT, galK, galM	
enterobacterales	Enterobacteriaceae	Enterobacter	cloacae	1	yes	no	yes	galT, galK, galM	
enterobacterales	Enterobacteriaceae	Salmonella	enterica ATCC 8400	1	yes	no	yes	galT, galK, galM	
enterobacterales	Enterobacteriaceae	Shigella	flexneri NCTC 9728	1	yes	no	yes	galT, galK, galM	

no galE in genome:	Xylella	fastidiosa
	Chromobacterium	violaceum
	Legionella	pneumophila pascullei NCTC12273

1. Schwengers O, Jelonek L, Dieckmann MA, Beyvers S, Blom J, Goesmann A. 2021. Bakta: rapid and standardized annotation of bacterial genomes via alignment-free sequence identification. Microbial Genomics 7:000685.

2. Bittar TB, Passow U, Hamaraty L, Bidle KD, Harvey EL. 2018. An updated method for the calibration of transparent exopolymer particle measurements. Limnology and Oceanography: Methods 16:621–628.
